# Steroids originating from bacterial bile acid degradation affect *Caenorhabditis elegans* and indicate potential risks for the fauna of manured soils

**DOI:** 10.1038/s41598-019-47476-y

**Published:** 2019-07-31

**Authors:** M. N. Mendelski, R. Dölling, F. M. Feller, D. Hoffmann, L. Ramos Fangmeier, K. C. Ludwig, O. Yücel, A. Mährlein, R. J. Paul, B. Philipp

**Affiliations:** 1 Institute of Zoophysiology, University of Münster (WWU), Münster, Germany; 2 Institute of Molecular Microbiology and Biotechnology, University of Münster (WWU), Münster, Germany; 3 Present Address: Institute for Pharmaceutical Microbiology, University Hospital Bonn, University of Bonn, Bonn, Germany

**Keywords:** Ecophysiology, Metabolism

## Abstract

Bile acids are steroid compounds from the digestive tracts of vertebrates that enter agricultural environments in unusual high amounts with manure. Bacteria degrading bile acids can readily be isolated from soils and waters including agricultural areas. Under laboratory conditions, these bacteria transiently release steroid compounds as degradation intermediates into the environment. These compounds include androstadienediones (ADDs), which are C_19_-steroids with potential hormonal effects. Experiments with *Caenorhabditis elegans* showed that ADDs derived from bacterial bile acid degradation had effects on its tactile response, reproduction rate, and developmental speed. Additional experiments with a deletion mutant as well as transcriptomic analyses indicated that these effects might be conveyed by the putative testosterone receptor NHR-69. Soil microcosms showed that the natural microflora of agricultural soil is readily induced for bile acid degradation accompanied by the transient release of steroid intermediates. Establishment of a model system with a *Pseudomonas* strain and *C*. *elegans* in sand microcosms indicated transient release of ADDs during the course of bile acid degradation and negative effects on the reproduction rate of the nematode. This proof-of-principle study points at bacterial degradation of manure-derived bile acids as a potential and so-far overlooked risk for invertebrates in agricultural soils.

## Introduction

Manure is known for containing hormonally active steroid compounds that may affect animal life in highly fertilized agricultural areas^1^. Apart from hormonally active steroids like estrogens, manure also contains further steroid compounds such as bile acids^2–4^. Bile acids are steroid compounds derived from cholesterol, which are produced in the liver of vertebrates and serve as detergents for the solubilisation of water-insoluble nutrients in the intestine and as signalling molecules^5–7^. Although 90% of bile acids are recycled in the so-called enterohepatic cycle, a considerable amount of bile acids is excreted via faeces and urine, in case of humans 400–600 mg per person and day^8^. Consequently, high amounts of bile acids can also be found in manure^9,10^. Cattle faeces contain 1 mg cholic acid and 3 mg deoxycholic acid per g and chicken faeces contain about 7.5 mg chenodeoxycholic acid (CDC) per g^2^. Dissolved in 1 ml water, this would yield 2 mM cholic acid and 7 mM deoxycholic acid or even 19 mM CDC for cow or chicken manure, respectively. In run-off simulations of poultry manure, about 0.3 mM deoxycholic acid and even 0.5 mM CDC were found^9^. In studies using bile acids as indicators for faecal contamination of rivers, up to 13 nM lithocholic acid and 76 nM deoxycholic acid were detected despite the high dilution in these environments^10^.

Upon excretion, bile acids are subject to bacterial degradation and transformation, and bile-acid degrading bacteria can easily be isolated from many environments^11–13^. The best-studied degradation pathway for bile acids is the so-called 9,10-*seco*-pathway of steroid degradation via Δ^1,4^-3-keto-intermediates^14–17^. Degradation is initiated by oxidation of the A-ring and splitting off the C_5_-carboxylic side chain yielding C_19_ steroids with Δ^1,4^-3,17-diketo structure^14,15,18,19^, which are called androstadienediones (ADDs). ADDs are also central intermediates in the degradation of other steroids such as cholesterol and testosterone^17,20^ (Supplemental Fig. S1).The further degradation of ADDs proceeds by oxygenation of C9, which results in cleavage of the B-ring^21,22^. These so-called *seco*-steroids are further degraded to structures consisting of only the C- and D-rings, which are then broken down to central intermediates of energy metabolism^23^.

A characteristic feature of bile acid degradation in laboratory cultures is the transient extracellular accumulation of several intermediates such as ADDs^11,12,24^. As ADDs have androgenic effects on mammals^25,26^ and are related to the biosynthesis of testosterone and estrogens^27,28^, their unusually high release into the environment due to massive input of manure may have hormonal effects. Such effects have been proposed for fish populations from rivers receiving phytosterol-containing wastewaters from paper mills^29–31^. In addition, invertebrates, such as molluscs and nematodes, were reported to be affected by vertebrate-like steroid hormones^32–35^.

The high abundance and diversity of nematodes and their manifold interactions in many food webs make nematode assemblages useful indicators of ecosystem conditions^36,37^. *Caenorhabditis elegans* is a free living, non-parasitic nematode, which is particularly abundant in microbe-rich terrestrial environments, especially rotting plant matter^38^. It is not only an important model organism for molecular biology or pharmacology^39^ but also an environmental indicator for investigating anthropogenic influences on ecosystems^39–44^.

Concerning the possibility of hormonal control by steroids, the genome of *C*. *elegans* is predicted to encode 284 nuclear hormone receptors (NHRs), with the human or fly genome encoding only 48 or 21 of them, respectively^45,46^. However, the ligands of *C*. *elegans* NHRs are yet unknown, with the exception of the steroid hormone receptor DAF-12^47^. In contrast to wild type, where the application of testosterone altered behavioural responses of *C*. *elegans* (e.g. the gentle touch response), such effects of testosterone were not detected in the outcrossed *nhr*-*69* (*ok1926*) deficient mutant strain^48^. As NHR-69 also showed the best matching sequence with the human androgen receptor (AR) ligand-binding domain, it was suggested that NHR-69 is a putative testosterone receptor and *nhr*-*69* a putative ortholog of the human AR gene. Another report^49^ additionally showed the capacity of NHR-69 to bind testosterone and also provided evidence that NHR-14 is an estrogenic hormone receptor. Steroid hormone synthesis requires cholesterol. *C*. *elegans*, however, is cholesterol auxotrophic, which means that cholesterol must be absorbed with the feed (e.g., yeast/plant leftovers, animal faeces)^50,51^. Ingested cholesterol is then used, *inter alia*, for the synthesis of ∆^4^- and ∆^7^-dafachronic acid (DA), which are the two known bile acid-like steroids that bind to DAF-12^52^.

Given the fact of an overfertilization of arable soil by manure, this study aimed at an investigation of the bacterial degradation products of bile acids (i.e., ADD intermediates) and their effects on invertebrates from microbe-rich terrestrial environments, using *C*. *elegans* as an intensely studied representative.

## Results

### Effects of testosterone and ADDs on the gentle touch response of wild type and *nhr*-*69∆*

**Figure 1:**
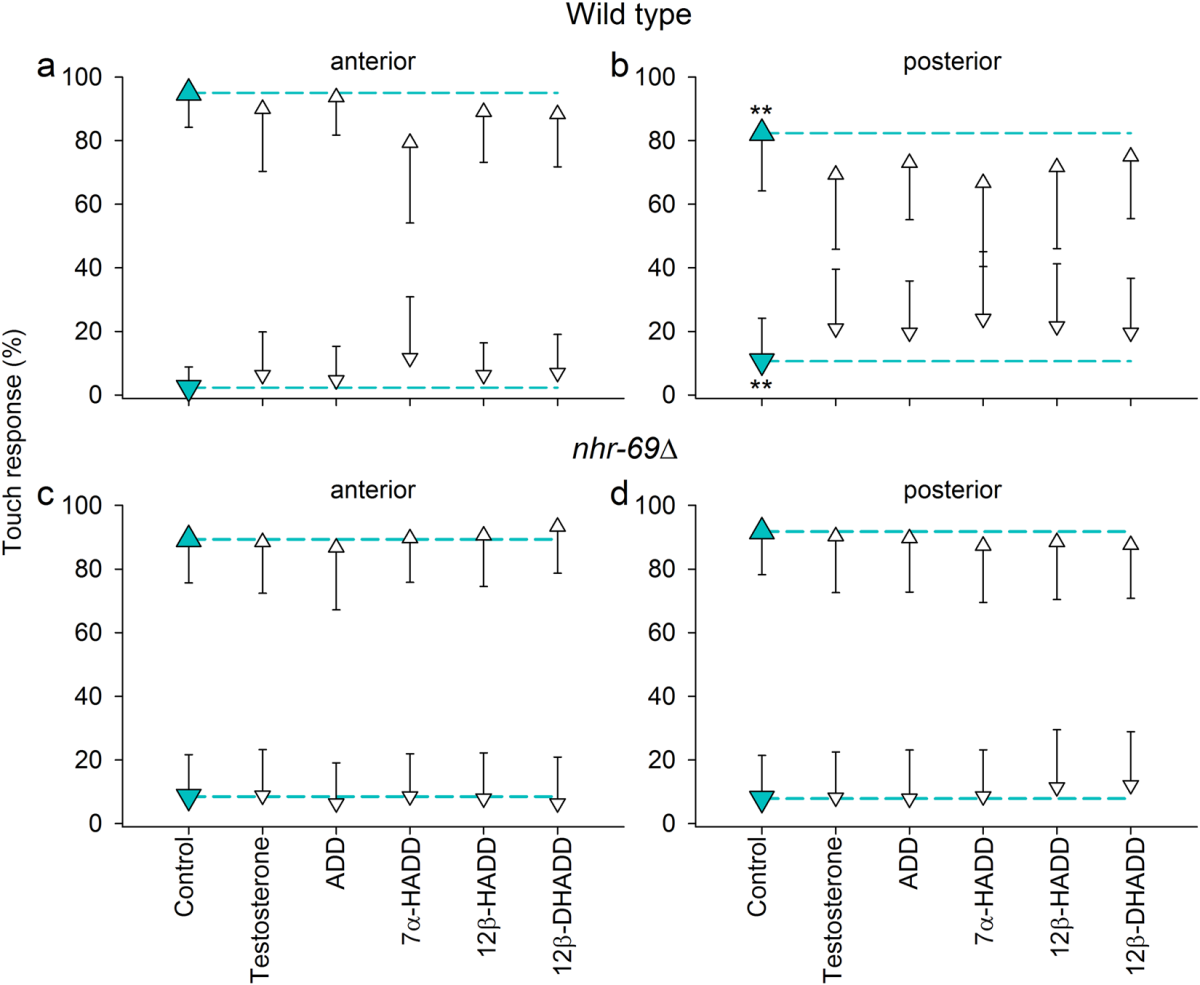
Effects of steroid compounds on the gentle touch response of wild type and *nhr*-*69∆*. Positive (upturned triangles) or negative reactions (downturned triangles) of *C*. *elegans* (**a**,**b**) wild type or (**c**,**d**) *nhr*-*69*∆ worms to successive tactile stimuli applied either to the (**a**,**c**) head (anterior) or (**b**,**d**) tail region (posterior) of L4 worms that had developed from egg to this larval stage under control (green symbols and lines) or five test conditions (exposures to testosterone, ADD, 7α-HADD, 12β-HADD, or 12β-DHADD) (mean ± sd; per experimental condition, *n* ≥ 60 different worms). Asterisks indicate significance levels (**P < 0.01; two-way anova and subsequent Student-Newman-Keuls analysis).

*Caenorhabditis elegans* wild type (Fig. 1a,b) or *nhr*-*69*∆ (*nhr*-*69* (*ok1926*) deficient mutant) (Fig. 1c,d) developed from egg to the late L4 larval stage under control or test conditions (exposure to 5 μM of testosterone, ADD, 7α-HADD, 12β-HADD, and 12β-DHADD). Applying ten successive tactile stimuli to the anterior (*a*) or posterior (*p*) region of the L4 worms and counting their positive (*a*, backward movement; *p*, forward movement) or negative (*a*, forward movement; *p*, backward movement) reactions revealed in case of posteriorly stimulated wild type significant decreases in positive and increases in negative reactions under test conditions in comparison to the control condition. These effects were not observed in case of *nhr*-*69*∆.

### Effects of testosterone and ADDs on the reproduction rate of wild type and *nhr*-*69∆*

**Figure 2:**
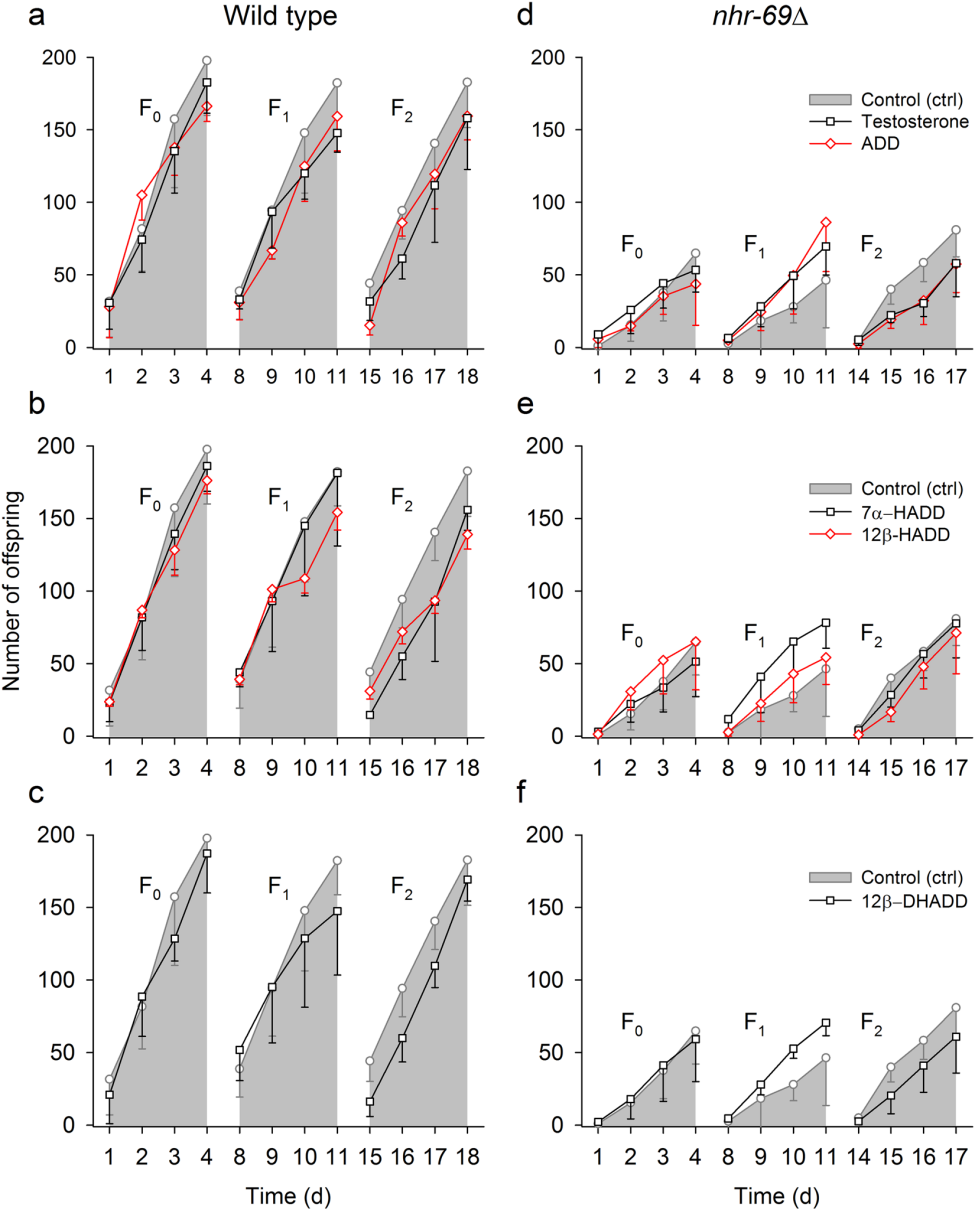
Effects of steroid compounds on the reproduction rate of wild type and *nhr*-*69∆*. Time-dependent increases in the number of offspring of individual *C*. *elegans* worms determined over three successive generations (F0, F1, F2) and over 3-day periods each in (**a**–**c**) wild type or (**d**–**f**) *nhr*-*69*∆ under control (grey symbols, lines, and areas) or five test conditions [exposures to (**a**,**d**) testosterone or ADD, (**b**,**e**) 7α-HADD or 12β-HADD, or (**c**,**f**) 12β-DHADD], with the F0 generation originating from worms still bred under the control condition (mean ± sd; per experimental condition and generation, *n* = 4–6 (wild type) or 3–5 (*nhr*-*69*∆) NGM plates with one egg laying worm each). Three-way anova and subsequent Student-Newman-Keuls analysis (see Supplemental Table S3, which summarizes all statistical analyses and significances for this figure) revealed in wild type and in comparison to the control condition negative effects of (**a**) testosterone or ADD, (**b**) 7α-HADD or 12β-HADD, or (**c**) 12β-DHADD on the number of offspring determined over all three generations, but no significant effects of these steroid compounds on the low number of offspring in *nhr*-*69*∆.

As a previous study^53^ has shown negative effects of testosterone on the fecundity of *C*. *elegans*, with this effect increasing significantly during the long-term exposure over several generations, wild type (Fig. 2a–c) or *nhr*-*69*∆ (Fig. 2d–f) were kept over three successive generations (F0, F1, F2) under control or test conditions (exposure to 5 μM of testosterone, ADD, 7α-HADD, 12β-HADD, and 12β-DHADD), with the F0 generation originating from worms still bred under the control condition.

Within each generation, the time-dependent increase in the number of offspring from individual worms was determined over 3-day periods. The number of offspring was consistently lower in *nhr*-*69*∆ worms. The number of offspring determined over all three generations was negatively affected in wild type, but not in *nhr*-*69*∆ by testosterone or ADD (Fig. 2a), 7α-HADD or 12β-HADD (Fig. 2b), or 12β-DHADD (Fig. 2c) in comparison to the control condition. Additionally, there were intergenerational differences in reproduction rate between F0 or F1 and F2 in wild type and *nhr*-*69*∆ in case of 7α-HADD and 12β-HADD exposure (Fig. 2b,e).

### Effects of testosterone and ADDs on the developmental speed of wild type and *nhr*-*69∆*

**Figure 3:**
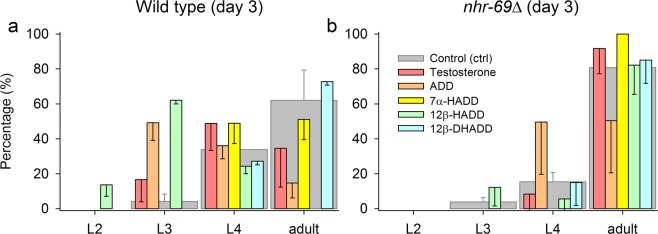
Effects of steroid compounds on the developmental speed of wild type and *nhr*-*69∆*. The developmental speed of *C*. *elegans* (**a**) wild type or (**b**) *nhr*-*69∆* was assessed under control or five test conditions (exposures to testosterone, ADD, 7α-HADD, 12β-HADD, or 12β-DHADD) by determining the percentage share of larval stages (L1–L4) and adult worms. Significant differences in developmental speed were detected three days after egg laying (mean ± se; per experimental condition, *n* = 3–4 NGM plates with one worm each, which was present only during egg laying). One-way anova and subsequent Student-Newman-Keuls analysis revealed significant differences in developmental speed between (**a**) wild type and (**b**) *nhr*-*69*∆ (*P* = 0.01) and in (**a**) wild type generally between all six conditions (*P* = 0.013) and specifically between 12β-HADD and 12β-DHADD treatment (*P* = 0.018) or 12β-HADD treatment and control condition (*P* = 0.035), with an almost significant difference between 12β-DHADD and ADD treatment (*P* = 0.051). There were no significant differences between the different conditions in *nhr*-*69*∆.

The developmental speed of *C*. *elegans* wild type (Fig. 3a) or *nhr*-*69∆* (Fig. 3b) from egg to adult worm was assessed under control or test conditions (exposure to 5 μM of testosterone, ADD, 7α-HADD, 12β-HADD, 12β-DHADD) by determining the percentage share of L1, L2, L3, and L4 larval stages and adult worms one, two, and three days after egg laying. Significant differences in developmental speed were detected on the third day. Wild type showed a significantly slower developmental speed than *nhr*-*69∆* as well as significant differences in developmental speed between different treatments (e.g. 12β-HADD exposure vs. 12β-DHADD exposure or the control condition).

### Effects of 7α-HADD on the transcriptome of wild type

**Figure 4:**
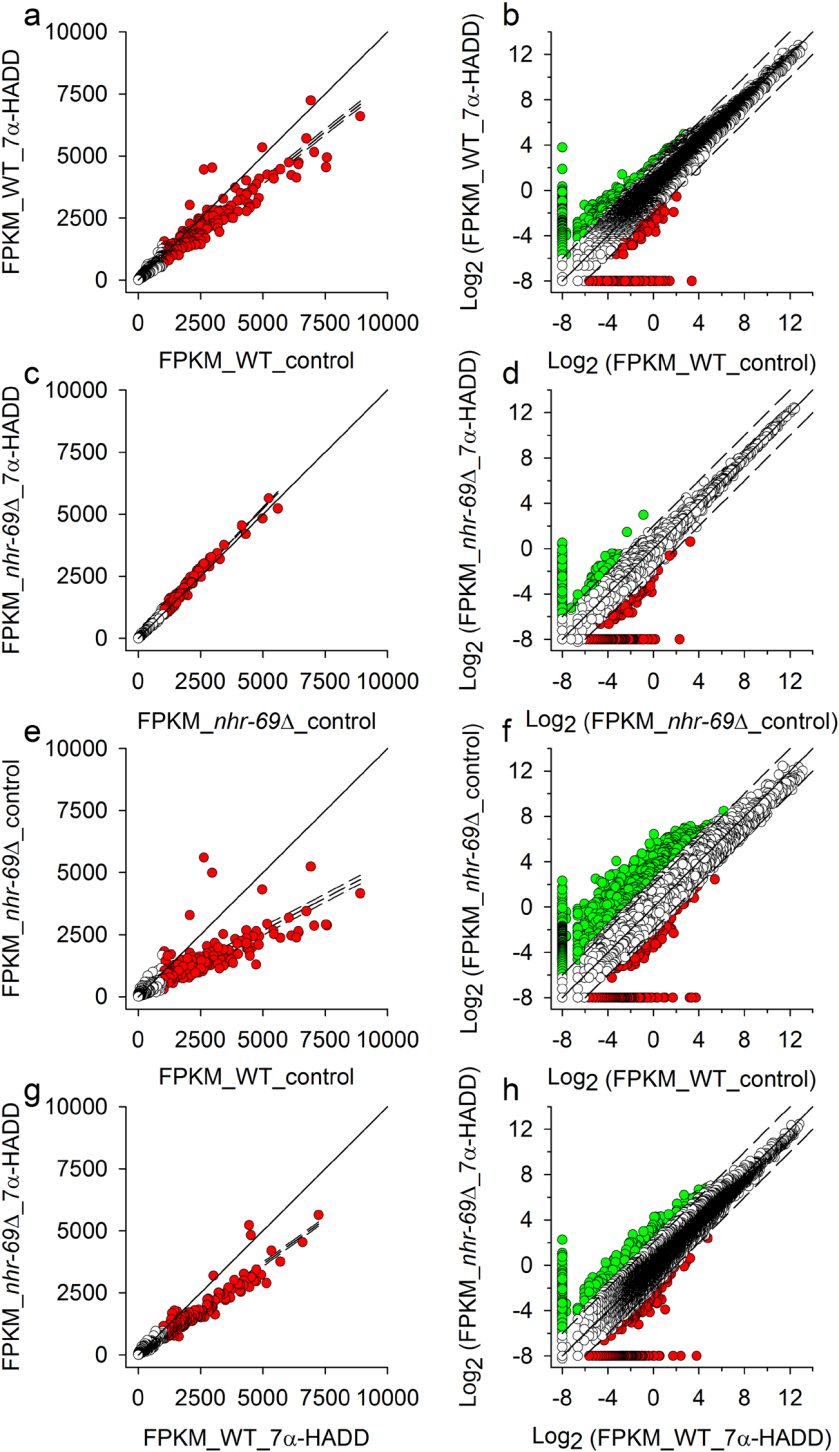
Gene expression intensities in wild type or *nhr*-*69*Δ after breeding under control or test conditions. Transcriptome profiling was performed by RNA-Seq to determine gene expression intensities in synchronized adult *C*. *elegans* (**a**,**b**) wild type (WT) or (**c**,**d**) *nhr*-*69*∆ that had developed from the L1 to the adult stage under control and test (7α-HADD exposure) conditions. Additionally, the gene expression intensities of the two *C*. *elegans* strains are shown under (**e**,**f**) control or (**g**,**h**) test conditions [averaged FPKM values; per strain and experimental condition, *n*: mostly 3 biological replicates (*see* Material and Methods)]. The x-y plots show the expression intensities of all genes, which carried a WormBase identification number (WB GeneID), as double linear (left graphs) or double log_2_ (right graphs) plots (continuous lines mark the 45-degree diagonal lines) to emphasize genes with higher or lower expression intensity. Genes with a mean FPKM value higher than 1000 on the x-axis mostly deviated from the 45-degree diagonal line (left graphs, red circles and dashed linear regression and 99% prediction lines). Genes with lower mean FPKM values and deviations from equal log_2_-fold expression intensity (45-degree diagonal lines) of lesser than −2 or greater than 2 between x-axis and y-axis (right graphs, green or red circles and dashed offsets of −2 and 2) were also functionally characterized by GO analyses (see Supplemental Table S1).

To explore the genetic basis of the described effects of ADDs in a case study, transcriptome profiling by RNA-Seq was carried out on synchronized adult *C*. *elegans* wild type or *nhr*-*69* that had developed from the L1 to the adult stage under control or test conditions (exposure to 5 μM of 7α-HADD). Within each of the ten used animal samples, more than 15,000 identified (i.e. with a WormBase identification number, WB GeneID) and expressed genes were detected, whose expression level was quantified using the FPKM method (see Material and Methods). Screening the differently regulated genes of the four different contrasts by the NOISeq method (see Material and Methods) revealed 1165 (WT_7α-HADD vs. WT_control), 3 (*nhr*-*69*∆_7α-HADD vs. *nhr*-*69*∆_control), 5386 (*nhr*-*69*∆_control vs. WT_control), and 4002 (*nhr*-*69*∆_7α-HADD vs. WT_7α-HADD) differentially expressed genes with a diverge probability of greater than or equal to 0.8 (abbreviated as DEGs). For analyzing differential gene regulation either for the greater number of genes with identical WB GeneID or the smaller number of DEGs, the mean expression intensities (averaged FPKM values) of corresponding genes or DEGs were plotted against each other (x-y plots) either in case of wild type (Fig. 4a,b; Supplemental Fig. S2a,b) or *nhr*-*69*∆ (Fig. 4c,d; the only three DEGs were not graphically represented) under control and test conditions or for wild type and *nhr*-*69*∆ under control (Fig. 4e,f; Supplemental Fig. S2c,d) or test (Fig. 4g,h; Supplemental Fig. S2e,f) conditions. As criteria for actually existing differences in gene regulation either distinct deviations from the 45-degree diagonal line in double linear plots (left graphs) or strong log_2_-fold differences in expression intensity in double log_2_ plots (right graphs) were chosen, with the two scale types chosen to emphasize genes with higher (double linear plots) or lower expression intensity (double log_2_ plots). The function of these gene groups was studied by gene ontology (GO) analyses (functional annotation chart; David Bioinformatics Resources 6.8). Most of these genes were functionally assignable.

The gene groups identified in the double linear plots (Fig. 4 and Supplemental Fig. S2, left graphs, red circles) commonly comprised genes or DEGs for translational processes, with annotation terms like ribosomal protein or translation (Supplemental Tables S1, S2). The same was valid for the hardly regulated genes of *nhr*-*69*∆ under control and test conditions (Fig. 4c). Thus, the varying degree of deviation from the 45-degree diagonal line indicates differences in the regulation of genes or DEGs for translational processes. Upregulations of genes or DEGs seemed to increase in the following order: *nhr*-*69*∆ under test conditions (Fig. 4c), wild type under control conditions (Fig. 4a; Supplemental Fig. S2a) or wild type in comparison to *nhr*-*69*∆ under test conditions (Fig. 4g; Supplemental Fig. S2e), and wild type in comparison to *nhr*-*69*Δ under control conditions (Fig. 4e; Supplemental Fig. S2c). Thus, gene expression for translational processes was higher in wild type than in *nhr*-*69*∆, and the exposure to 7α-HADD reduced this type of gene expression in wild type but affected it hardly (genes) or not (DEGs) in *nhr*-*69*∆.

The groups of genes identified in the double log_2_ plots (Fig. 4, right graphs, green and red circles) comprised genes, which were frequently assigned to terms that are likely related to (i) steroid hormones (e.g., NHRs) (Supplemental Table S1, red background colour), (ii) G protein-coupled receptors (GPCRs) and sensory perception (e.g., serpentine receptors) (Supplemental Table S1, yellow background colour), (iii) innate immune response (e.g., C-type lectins) (Supplemental Table S1, green background colour), and (iv) phosphorylation/dephosphorylation (e.g., protein kinases) (Supplemental Table S1, blue background colour).

**Figure 5:**
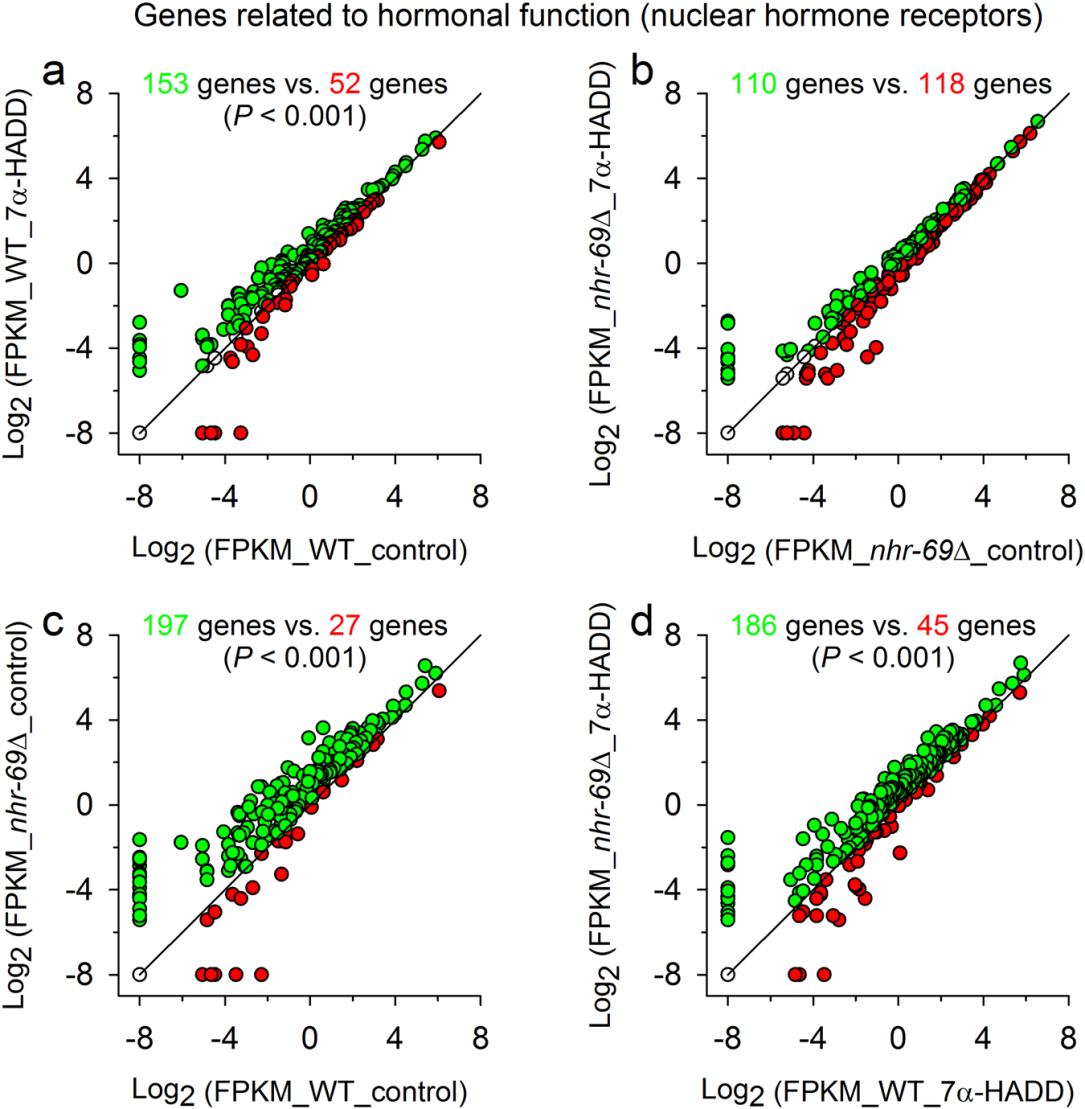
Expression intensities of genes related to hormonal function in wild type or *nhr*-*69*Δ after breeding under control or test conditions. Double log_2_ plots of the expression intensities (mean FPKM values) of genes related to hormonal function in synchronized adult *C*. *elegans* (**a**) wild type (WT) or (**b**) *nhr*-*69*∆ bred from egg to adult worm under control and test (7α-HADD exposure) conditions. Additionally, the gene expression intensities of the two *C*. *elegans* strains are shown under (**c**) control or (**d**) test conditions. Deviating regulation (i.e., up- or downregulated genes) is indicated by different symbol colours (green or red circles). The numbers of up- or downregulated genes are given in the corresponding colour, together with the significance level (*P* values from chi-square analyses) in case of significantly deviating regulation (see text). Continuous lines mark the 45-degree diagonal lines.

**Figure 6:**
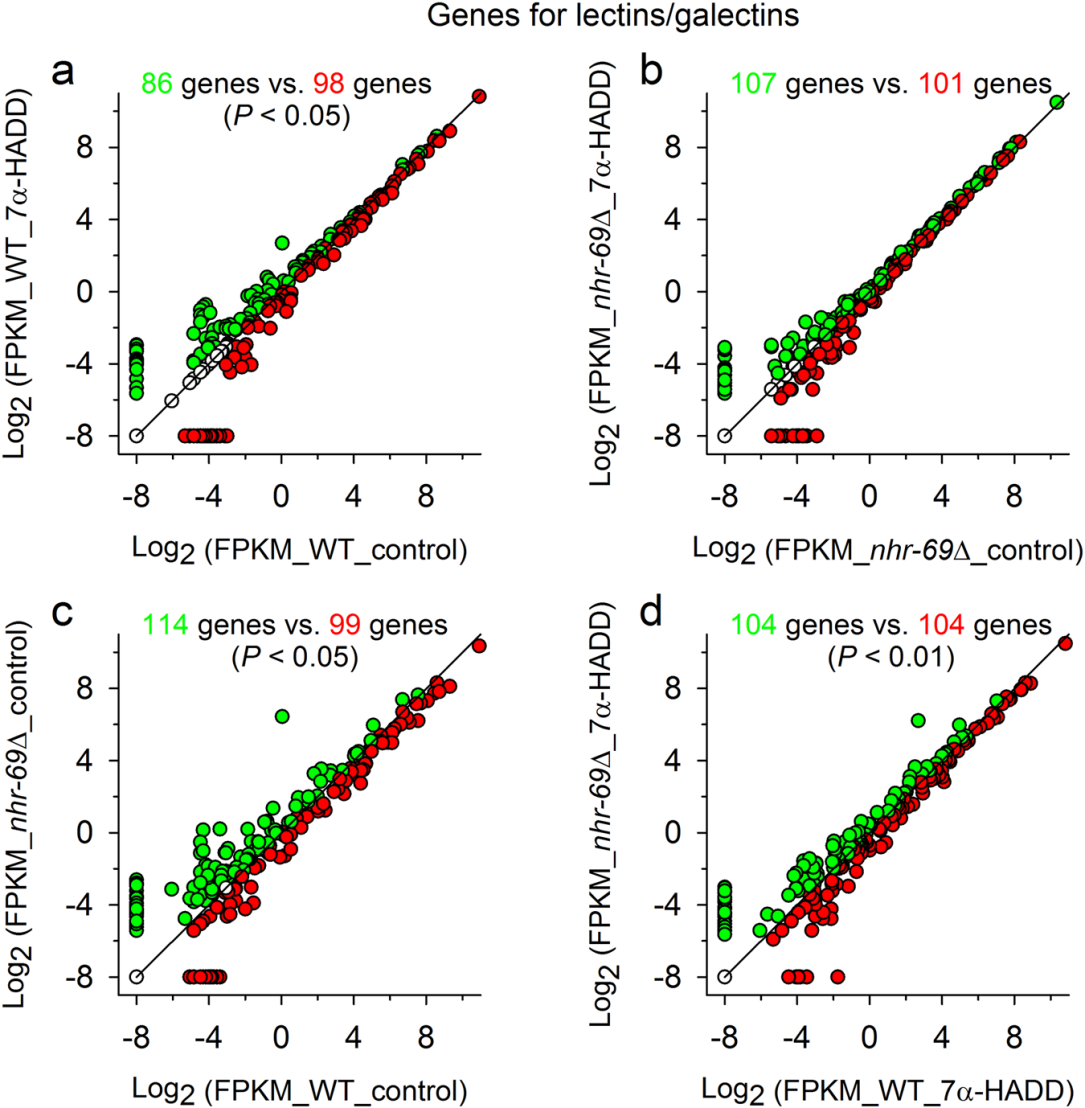
Expression intensities of genes for lectins or galectins in wild type or *nhr*-*69*Δ after breeding under control or test conditions. Double log_2_ plots of the expression intensities (mean FPKM values) of genes for lectins or galectins in synchronized adult *C*. *elegans* (**a**) wild type (WT) or (**b**) *nhr*-*69*∆ bred from egg to adult worm under control and test (7α-HADD exposure) conditions. Additionally, the gene expression intensities of the two *C*. *elegans* strains are shown under (**c**) control or (**d**) test conditions. Deviating regulation (i.e., up- or downregulated genes) is indicated by different symbol colours (green or red circles). The numbers of up- or downregulated genes are given in the corresponding colour, together with the significance level (*P* values from chi-square analyses) in case of significantly deviating regulation (see text). Continuous lines mark the 45-degree diagonal lines.

Thus, we screened the results from the Basic Local Alignment Search Tool (BLAST), which was applied on the RNA-Seq data by the Beijing Genomics Institute (BGI), for corresponding genes and detected numerous genes for hormonal functions (essentially genes for NHRs), serpentine receptors, and lectins or galectins. The mean expression intensities of these genes were then analysed in wild type or *nhr*-*69*∆ under control and test conditions in a similar way as aforementioned (x-y double log_2_ plots; Fig. 4), with the difference that a distinction was now made between up- or downregulated genes and that gene enrichment analyses (chi-square tests) were carried out to identify deviating regulation, for which the gene-specific ratio between up- and downregulated genes (e.g., genes for hormonal functions) was significantly different from the contrast-specific ratio (e.g., wild type under control and test conditions). These analyses showed in case of genes for hormonal functions significant upregulations in wild type due to 7α-HADD exposure (Fig. 5a) and significant upregulations in *nhr*-*69*∆ in comparison to wild type under control (Fig. 5c) or test conditions (Fig. 5d). Deviating regulation was not detected in case of genes for serpentine receptors. Genes for lectins or galectins showed significant upregulations (in relation to the contrast-specific ratio) in wild type either under control conditions (Fig. 6a) or in comparison to *nhr*-*69*∆ under control (Fig. 6c) or test (Fig. 6d) conditions.

The groups of DEGs identified in the double log_2_ plots (Supplemental Fig. S2, right graphs, green and red circles) comprised genes for quite different processes such as developmental (Supplemental Table S2, yellow background color) or nuclear processes including DNA damage and repair (Supplemental Table S2, blue background color). Genes for developmental processes seemed to be upregulated in wild type under test conditions or in comparison to *nhr*-*69*∆ under control conditions. Genes for nuclear processes were found to be upregulated in wild type under test conditions and in both strains under control or test conditions.

### CDC degradation in soil microcosms

The presented results with isolated steroids from bacterial bile acid degradation raised the question whether such effects could also be observed when natural habitats of nematodes are supplied with bile acids, which are degraded by the endogenous bacteria. This investigation would in particular be interesting if the extracellular accumulation of intermediates of bile acid degradation could also be detected in the pore water of soil microcosms.

**Figure 7:**
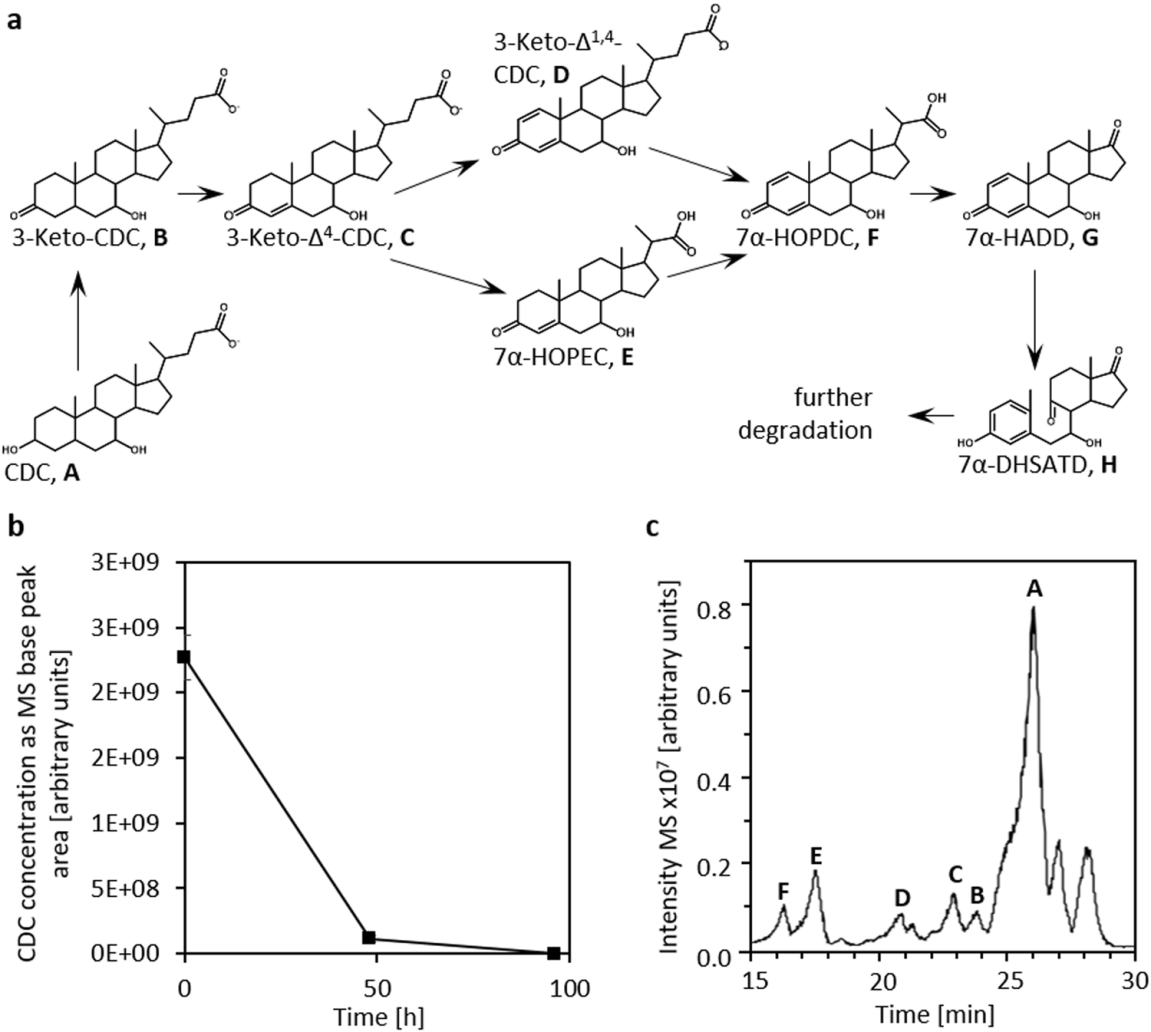
Fate of CDC in soil slurries. (**a**) Overview over CDC degradation by *P*. *stutzeri* Chol1. 7α-HOPDC: Hydroxy-3-oxo-pregna-Δ^1,4^-diene-carboxylate, 7α-DHSATD: 7α-Dihydroxy-9,10-*seco*-androsta-Δ^1,3,5^-triene-9,17-dione, (**b**) Degradation of 1 mM CDC in soil slurry. CDC concentration was determined as base peak area of total ion counts in negative mode MS measurements. (mean ± sd, *n* = 3). (**c**) MS base peak chromatogram of the extracted supernatant of a soil slurry incubated with 1 mM CDC for about 48 h and proposed structures of degradation intermediates. Samples were measured in negative MS mode; structure assignments are based molecular masses and UV absorption spectra.

As 7α-HADD showed strong effects on the physiology of *C*. *elegans*, further investigations were focused on CDC as its precursor in bacterial degradation. For investigating whether CDC is degraded in soil, slurry experiments were conducted with soil collected near agriculturally used fields in the Münsterland region. In all experiments, 1 mM CDC was completely degraded within about one week (Fig. 7a,b). Especially after 2 d, many intermediates were detectable, which were identified as 3-keto-CDC, Δ^4^-3-keto-CDC, 7α- hydroxy-3-oxo-pregna-Δ^1,4^-diene-carboxylate (7α-HOPDC) and the Δ^4^-monoene corresponding to 7α-HOPDC (Fig. 7c) by UV-spectroscopy and mass spectrometry as described earlier^54^ These steroid metabolites are also found in supernatants of laboratory cultures of bile acid degrading bacteria such as *Pseudomonas stutzeri* Chol1^11,54^.

### CDC degradation in sand microcosms and effects on *C*. *elegans*

In a next step, it was tested whether the reproduction of *C*. *elegans* is affected by the presence of bile acid degrading bacteria. However, a reliable quantification of *C*. *elegans* individuals in soil microcosms was not possible. Nevertheless, the reproduction of *C*. *elegans* can be quantified in semi-natural sand microcosms with *E*. *coli* as food bacteria^55^. For enabling bacterial bile acid degradation, the model organism *P*. *stutzeri* Chol1 was additionally used here^54^.

In preliminary experiments on sand microcosms without worms but with 0.25 mM CDC for testing CDC degradation, a transient extracellular accumulation of 3-keto-CDC, 7α-HOPDC, 7α-HADD and the *seco*-steroid 7α-dihydroxy-9,10-*seco*-androsta-Δ^1,3,5^-triene-9,17-dione (7α-DHSTAD) was observed (Supplemental Fig. S3).

**Figure 8:**
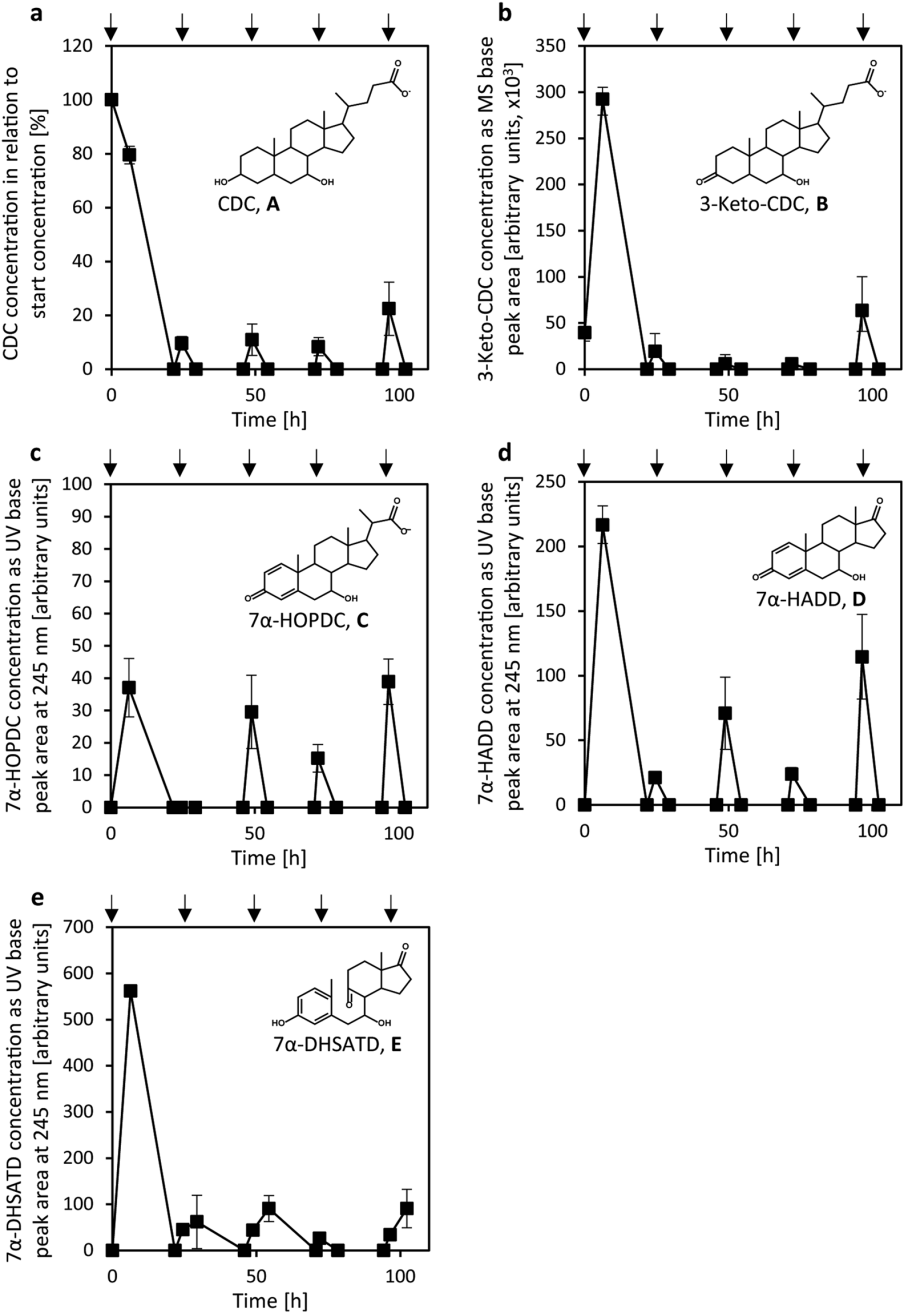
CDC degradation by *P*. *stutzeri* Chol1 in sand microcosms containing *E*. *coli* OP50_-ura_ but not *C*. *elegans*. Degradation kinetics of CDC (**a**), 3-keto-CDC (**b**), 7α-HOPDC (**c**), 7α-HADD (**d**) and 7α-DHSATD (**e**). Arrows indicate the addition of fresh suspensions of both bacteria and 0.25 mM CDC. For each sample, the content of one sand microcosm was sacrificed and analysed by LC-MS. CDC concentration was determined as base peak area of total ion counts in negative mode in relation to the start concentration. Concentration of 3-keto-CDC was determined as base peak area of total ion counts in negative mode MS measurements. Concentration of 7α-HADD and 7α-HOPDC were determined as base peak areas in UV chromatograms at a wavelength of 245 nm and 7α-DHSATD at a wavelength of 280 nm. (mean ± sd, *n* = 3).

As *C*. *elegans* incubations should last several days, bile acid degradation after repeated additions of CDC was determined (Fig. 8). About 20 h after each addition of CDC, CDC was completely degraded. About 6 h after each addition of CDC, the intermediates described above were detected and after 20 h, they were degraded. In the presence of *E*. *coli* OP50_-ura_ at an optical density at 600 nm (OD_600_) of 0.147, CDC was not degraded, but partially transformed to 7-keto-CDC (Supplemental Fig. S4). In order to test whether *C*. *elegans* has effects on CDC degradation, experiments were repeated with ten adult worms on each plate. In these preparations, CDC degradation took place as in the preparations without *C*. *elegans* (Supplemental Fig. S5).

**Figure 9:**
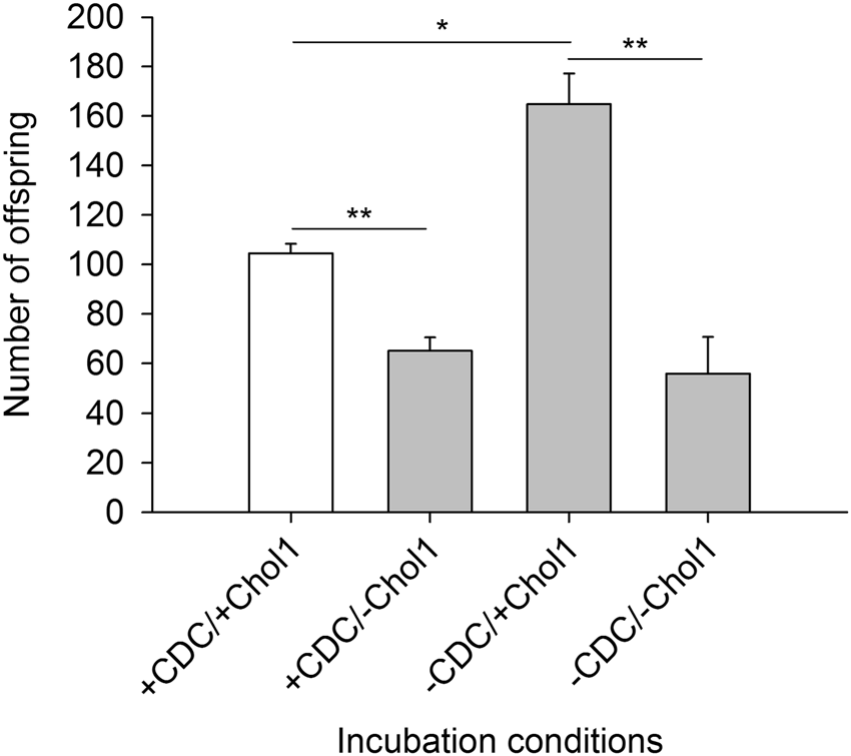
Steroid compounds from CDC degradation by *P*. *stutzeri* Chol1 reduced the reproduction rate of wild type. Number of offspring (normalized values; *see* Material and Methods) of adult *C*. *elegans* worms after four days of incubation with (+) or without (−) CDC or *P*. *stutzeri* Chol1 (mean ± se; per experimental condition, *n* = 3–4 biological replicates, and per biological replicate, *N* = 11 petri dishes, with each petri dish containing one egg laying worm within a semi-natural medium). Asterisks indicate significance levels (*P < 0.05, **P < 0.01; t-test).

Finally, the reproduction rate of adult *C*. *elegans* worms was quantified in sand microcosms (Fig. 9). The presence or absence of CDC without *P*. *stutzeri* Chol1 showed no significant effects on reproduction rate. The addition of *P*. *stutzeri* Chol1 to the already present food bacteria (*E*. *coli* OP50_-ura_) promoted the reproduction rate of *C*. *elegans* which, however, was reduced by the addition of CDC.

## Discussion

This study aimed at an investigation of possible effects of ADDs, which are formed as intermediates of bile acid degradation by bacteria, on the nematode *Caenorhabditis elegans*. Three primarily unrelated observations initiated this study, namely: (1) all bile-acid degrading bacteria analysed so far release steroid intermediates during growth in laboratory cultures^11^, (2) bile-acid degrading bacteria are widespread and can readily be isolated from soil and freshwater environments^11^, and (3) high loads of manure lead to an unusual high input of bile acids on agricultural land^2^. As ADDs are among the most prominent steroid intermediates transiently released during bacterial bile acid degradation and are known to be hormonally active compounds, the degradation of bile acids might lead to endocrine effects on the soil fauna in manured soils. Our proof-of-concept study clearly showed that the tested ADDs, which were derived from four of the main bile acids occurring in manure (e.g., 7α-HADD), have multiple effects on essential traits of *C*. *elegans* when applied in environmentally relevant concentrations. As these effects were absent in *nhr*-*69*∆, it is possible that the tested steroids act on the NHR-69 receptor.

The developmental exposure of wild type to testosterone or ADDs negatively affected the posterior touch response, the reproduction rate in a transgenerational manner as well as the developmental speed of wild type but not of *nhr*-*69*∆ worms. Regarding the gentle touch response, Gámez-Del-Estal *et al*.^48^ have also reported fewer positive anterior or posterior touch responses in wild type after the exposure to testosterone (0.01–1 mM) during development. This effect lasted over four subsequent generations in the absence of the hormone. As the deacetylase inhibitor sodium butyrate or development over the Dauer stage eliminated the transgenerational effect of testosterone application, the authors suggested possible epigenetic effects of this hormone. BLAST searches showed the best matching of NHR-69 with the human androgen receptor ligand-binding domain, and effects of testosterone on the touch response were not detected in the outcrossed (2×) *nhr*-*69*∆ mutant strain [*nhr*-*69*(*ok1926*) I], which together suggest that NHR-69 can bind testosterone. Mimoto *et al*.^49^ also demonstrated the capacity of NHR-69 to bind testosterone. As specific neural circuits are responsible for the gentle touch response of *C*. *elegans*^56^, testosterone may have affected the functionality of neuronal synapses via NHR-69-mediated gene expression^48^, which may also apply to the ADDs tested in the present study.

Regarding the reproduction rate, Tominaga *et al*.^53^ have also shown a reduced fecundity of wild type after a developmental exposure to 5 μM of testosterone, with this effect increasing significantly during a long-term exposure over five generations. Thus, epigenetic effects may also contribute to the testosterone-mediated effects on reproduction rate (see above). The lower reproduction rate of *nhr*-*69*∆ and the absent effects of the tested steroid compounds on the fecundity of this strain in the present study may have resulted from absent positive effects of NHR-69 on reproduction, mediated by a yet unknown steroid hormone. The tested steroids, however, showed negative effects on the reproduction of wild type, possibly due to a competitive inhibition of the NHR-69 receptor.

The reason for the negative effects of the tested steroid compounds on the developmental speed of wild type may also be a competitive inhibition of NHR-69. As the developmental speed of *nhr*-*69*∆, however, was not significantly affected by the tested steroid compounds, the mutant may have involved other NHRs and steroid hormones in the determination of developmental speed, which possibly compensated for the missing NHR-69 receptor.

Thus, the tested steroid compounds may have negatively affected the NHR-69 receptor, which seems to be involved in reproduction and the gentle touch response but is obviously not obligatory in the determination of developmental speed.

Transcriptome profiling of the effects of 7α-HADD on *C*. *elegans* wildtype and *nhr*-*69*∆ supported these findings, as significant effects on gene expression for protein biosynthesis, hormone receptors, and proteins involved in the innate immune response were detected.

Gene expression for protein biosynthesis was higher in wild type than in *nhr*-*69*∆, and it decreased in wild type but remained almost unaffected in *nhr*-*69*∆ upon the exposure to 7α-HADD. An upregulated gene expression for protein biosynthesis indicates increasing investments in the translational machinery, with the aim of elevating the rate of protein biosynthesis. These proteins are required, for instance, for reproductive processes. Thus, the higher reproduction rate and upregulated gene expression for protein biosynthesis in wild type in comparison to *nhr*-*69*∆ as well as the lower reproduction rate and downregulated gene expression for protein biosynthesis in wild type upon the exposure to 7α-HADD are likely related to each other. The absent effects of 7α-HADD on the reproduction rate of *nhr*-*69*∆ and the hardly present effects of this steroid compound on gene expression for protein biosynthesis in the mutant strain also match this potential relation.

Genes for hormonal functions (mostly for NHRs) were upregulated in *nhr*-*69*∆ in comparison to wild type and upregulated in wild type upon the exposure to 7α-HADD. The upregulation of these genes in *nhr*-*69*∆ in comparison to wild type or in 7α-HADD-exposed wild type may reflect a compensatory mechanism for a missing or blocked NHR-69 receptor by an intensified expression of other NHRs, which are hardly or not affected by the tested steroid compound.

C-type lectins are secreted antimicrobials, and their genes are targets of the p38/PMK-1 mitogen-activated protein kinase (MAPK) pathway^57,58^. Genes for lectins or galectins were downregulated in *nhr*-*69*∆ in comparison to wild type and downregulated in wild type under test conditions. These findings indicate a suppression of the p38/PMK-1 MAPK pathway followed by a downregulated gene expression for lectins and galectins in case of a missing or blocked NHR-69 receptor. Thus, negative effects of an exposure to 7α-HADD on the innate immune response of wild type are possible.

Analysing specifically the expression of DEGs also showed the already discussed effects of 7α-HADD and/or strain on gene expression for protein biosynthesis (see above). Furthermore, DEGs for developmental processes seemed to be upregulated in wild type in comparison to *nhr*-*69*∆ under control conditions. However, the expression of these DEGs and DEGs for nuclear processes was evidently also higher in 7α-HADD-exposed wild type, which indicates effects of this steroid compound on still unclarified developmental processes. Most interestingly, differential gene expression was almost absent in 7α-HADD-exposed *nhr*-*69*∆ (i.e. 3 DEGs), which indirectly points to effects of this steroid compound on the NHR-69 receptor in wild type.

In soil and sand microcosms, CDC was readily degraded by natural microflora as well as by a model bacterium, which led to the transient release of degradation intermediates in both cases. Thus, an extracellular accumulation is not restricted to laboratory liquid cultures but occurs also during bile acid degradation in semi-solid environments. The reason for the transient extracellular accumulation of intermediates of bile acid degradation is still unknown. As bile acids and also part of their degradation intermediates are toxic to bacterial cells, it is likely related to the prevention of toxic effects^12,59,60^. Active export of bile acids via efflux pumps is well-known from gastrointestinal pathogens such as *Vibrio cholerae*^61^, which do not use bile acids for growth. Regarding steroid transport processes in bile acid degrading bacteria, respective mechanisms are only known so far in Gram-positive bile acid degrading bacteria of the genus *Rhodococcus*^24^. For Gram-negative Proteobacteria from which most of bile acid degrading bacteria originate, nothing has been published about steroid transport processes. ADDs were not detectable in the soil microcosms, which might be due to a strong adsorption of the relatively hydrophobic ADDs to soil particles, which has already been described for estradiol^62,63^. Nevertheless, ADDs may still be bioavailable to the nematodes. In the generally less hydrophobic sand microcosms, in contrast, an extracellular accumulation of 7α-HADD was observed at concentrations sufficient for causing effects in *C*. *elegans*.

In sand microcosms, the reproduction rate of worms was very variable and low with *E*. *coli* OP50_-ura_ as single food bacterium The presence of *P*. *stutzeri* Chol1 without CDC caused a higher number of offspring, which might be explained by the low quality of the standard food bacterium *E*. *coli* OP50_-ura_, as a previous study has already reported that the addition of little amounts of other bacterial species to *E*. *coli* can promote the development and reproduction of *C*. *elegans*^64^. The presence of *P*. *stutzeri* Chol1 with CDC, however, evidently reduced the number of offspring probably due to the negative effects of degradation products of CDC produced by *P*. *stutzeri* Chol1 on *C*. *elegans*.

Besides ADDs, many other steroid degradation intermediates accumulated in sand microcosms, among them Δ^1,4^-3-keto bile acid derivatives with a full C_5_-side chain as well as with a shortened C_3_-side chain. These compounds resemble DAs with known effects on *C*. *elegans*^65^. Additionally, 9,10-*seco*-steroids, which are also known to have biological activities^66^, were formed. Therefore, the impact of CDC degradation might not be restricted to ADDs and, thus, might have contributed to the higher variability of the reproduction rate of *C*. *elegans* in the sand microcosms.

In natural habitats, the diversity of bile acids and their bacterial degradation intermediates is certainly even larger. Apart from the well-described Δ^1,4^-3-keto-pathway^15,16,67,68^, there is also an alternative pathway for initiating bile acid degradation that proceeds via Δ^4,6^-3-keto intermediates, which also transiently accumulate outside the cells^69,70^. In this respect, it has been shown that cross-feeding between bacteria using these different pathways may result in dead-end intermediates^11^. Furthermore, anaerobic steroid degradation with nitrate as electron acceptor can give rise to further steroid degradation intermediates^71,72^ and would be plausible to occur in oxygen-depleted zones in manured soils. In conclusion, our study calls attention to potentially harmful effects on the mesofauna caused by the bacterial transformation of bile acids in habitats with a high impact of manure. The accumulation of ADDs can also arise from the degradation of sterols, which make up a higher fraction of sterols in manure^2,16^. In this respect, bacterial steroid transformation may be a so far overlooked factor contributing to the decreasing biodiversity in agricultural environments^73–75^. Experiments for investigating bacterial bile acid degradation under *in situ* conditions as well as specific feeding experiments are on the way in our laboratory.

## Material and Methods

### Cultivation of bacteria

*Escherichia coli* OP50_-ura_ was obtained from the Caenorhabditis Genetics Center (CGC; https://cgc.umn.edu/) and cultivated in LB medium (1% tryptone, 0.5% yeast extract, 1% NaCl)^76^ or OP50 medium (2% peptone, 0.5% NaCl, 0.001% uracil; pH 7.4). *Pseudomonas stutzeri* Chol1 was cultivated under aerobic and anaerobic conditions in the phosphate-buffered mineral medium MMChol with 1 mM of bile acids as carbon source^12^. Bacterial growth was monitored by measuring the OD_600_ with a spectrophotometer. For sand microcosms, 5 ml of *E*. *coli* OP50_-ura_ or *P*. *stutzeri* Chol1 cultures were harvested either in the late exponential or stationary growth phase by centrifugation (8,000 x *g*, 3 min, 20 °C) or after supplying 20% (v/v) fresh LB or 1 mM CDC to the cultures, respectively, and 1 h of further incubation. The cells were then washed and resuspended in MMChol without carbon source. New cell suspensions for sand experiments were prepared daily.

### Cultivation of worms

The N2 Bristol variety of *C*. *elegans* (wild type, WT) and the deletion mutant *nhr*-*69(ok1926)* I (*nhr*-*69*∆) were obtained from the CGC (https://cgc.umn.edu/). Worm cultures were maintained at 20 °C on nematode growth medium (NGM) plates with *E*. *coli* OP50_-ura_ in OP50 medium (OD_600_ = 1) as the food source^77^. During experiments, the NGM plates contained the minimum amount of cholesterol that supports *C*. *elegans* growth (0.5 μM of cholesterol)^53^. Control plates contained no further steroid compounds, whereas test plates contained 5 μM of either testosterone, ADD, 7α-HADD, 12β-HADD, or 12β-DHADD.

### Preparation of steroid compounds

Cholic acid (≥99%) from ox or sheep bile, deoxycholic acid (≥97%), lithocholic acid (≥98%), and testosterone were purchased from Sigma-Aldrich (St. Louis, MO, USA). CDC (≥98%) was purchased from Carl Roth GmbH + Co. KG (Karlsruhe, Germany), and ADD was purchased from Tokyo Chemical Industry UK Ltd. (Oxford, United Kingdom). For the preparation of 7α-HADD, 12β-HADD or 12β-DHADD, *P*. *stutzeri* Chol1 was grown under anoxic conditions, with 2 mM of deoxycholic acid, CDC or cholic acid as carbon source, respectively, as described^12,78^. All three ADDs were purified by organic extraction with dichloromethane^78^. The purity of steroid compounds was assessed by chromatography coupled to mass spectrometry and concentrations were determined photometrically^78^. For preparing stock solutions, testosterone and ADD were diluted in 100% ethanol, 7α-HADD and 12β-HADD in 70% ethanol, and 12β-DHADD in 50% ethanol. Cholic acid and CDC were diluted in MilliQ pure water (Merck, Darmstadt, Germany).

### LC-MS analysis

Steroid compounds and culture supernatants were analysed by liquid chromatography coupled to mass spectrometry (LC-MS) using a Dionex Ultimate 3000 LC system with a UV/visible light diode array detector (ThermoFisher Scientific; Waltham, MA, U.S.A.), and an ion trap mass spectrometer (Amazon speed; Bruker, Bremen, Germany) with an electro-spray ion source (ESI) as described previously^70^. For the evaluation of measurements, MS- or UV-base peak chromatograms or extracted ion chromatograms with defined mass ranges were used as indicated. For analysing the degradation of CDC and the accumulation of intermediates in sand, samples were centrifuged at >16,000 × *g* for 5 min at room temperature, before the supernatants were used for LC-MS analyses. For analysing the degradation of CDC and the accumulation of intermediates in the presence of *C*. *elegans*, samples were centrifuged as described, stored at −20 °C, acidified (pH 1–2), cleaned from hydrophilic contaminants by organic extraction with ethyl acetate, and dissolved in methanol. Samples from soil experiments were also cleaned by organic extraction prior to the analysis by HPLC-MS. For that, 200 μl of samples were acidified with 30 μl of 1 M of HCl (pH 1–2) and extracted with 600 μl of ethyl acetate. Ethyl acetate was dried off, and the samples were dissolved in 150 μl of ethanol. All extracted samples were then analysed by LC-MS.

### Gentle touch response assay

Single adult WT or *nhr*-*69∆* worms were allowed to lay eggs for 4 h at 20 °C on test and control NGM plates, before the adult worms were picked off these plates. After developing from egg to the late L4 larval stage at 16 °C, with *E*. *coli* OP50_-ura_ in OP50 medium as the food source, the gentle touch response of the L4 worms was tested. The worms were slightly touched ten times just behind the pharynx (anterior, *a*) or before the anus (posterior, *p*) using an eyebrow mounted on a glass pipette. Positive (*a*, backward movement; *p*, forward movement), negative (*a*, forward movement; *p*, backward movement), or ambiguous reactions were scored^56,79^.

### Fecundity assay

Single L4 worms of WT or *nhr*-*69∆* were transferred to test and control NGM plates with *E*. *coli* OP50_-ura_ in OP50 medium as the food source (T = 16 °C). The offspring of these worms (including the eggs) was counted every day over 3-day periods (F0 generation) and kept under these conditions for further breeding. Single L4 worms of WT or *nhr*-*69∆* from the F0 generation were then transferred to new test or control plates, and the offspring was again counted every day over 3-day periods (F1 generation). The same procedure was applied to determine the offspring of single L4 worms of WT or *nhr*-*69∆* from the F1 generation (F2 generation).

### Development assay

Single adult WT or *nhr*-*69∆* worms were allowed to lay eggs for 4 hours at 20 °C on test and control NGM plates, before the adult worms were picked off these plates. The developmental speed from egg to adult worm at 20 °C, with *E*. *coli* OP50_-ura_ in OP50 medium as the food source, was determined by counting the number of L1, L2, L3, L4 and adult worms after 1, 2, and 3 days.

### Transcriptome profiling

Synchronized^77^ L1 worms of WT or *nhr*-*69∆* developed to the adult stage on control or test (exposure to 5 µM of 7α-HADD) NGM plates with *E*. *coli* OP50_-ura_ in OP50 medium as the food source (T = 16 °C). Then, worms were washed from the plates and cleaned two times using purified water to minimize the number of bacteria. The worms from five NGM plates per strain and experimental condition were combined to one animal sample, and three animal samples per strain and experimental condition (i.e., 12 animal samples in total) were further prepared for transcriptome profiling by RNA-Seq as described^80,81^. RNA samples were sent to the BGI for RNA-Seq analysis. Using Illumina HiSeq2000 technology, the RNA samples were sequenced with a minimum of 10 mega reads per sample and a sequencing quality of more than 98% clean reads. Sequences were mapped to WormBase release WS257. Expression intensities of genes were calculated (see below) using the FPKM method (fragments per kilobase of exon per million fragments mapped) to normalize for sequencing depth and gene length^82^, which can be directly used for comparing differences in gene expression between samples. In case of WT under control or test conditions, the data from one animal sample each differed significantly from the two others regarding the expression intensity of corresponding genes, and mean expression intensities were calculated here using the data from only two animal samples. In case of *nhr*-*69*∆, however, the data from all three animal samples per experimental condition were useable for the calculation of mean expression intensities. Screening genes with differences in mean expression intensity in wild type or *nhr*-*69*∆ between control and test conditions or between both strains under control or test conditions by the NOISeq method^83^ revealed differentially expressed genes with a diverge probability of greater than or equal to 0.8 (termed DEGs). David Bioinformatics Resources 6.8 (functional annotation chart) was used for GO analyses.

### Bile acid degradation in soil microcosms

Soil microcosms were prepared by mixing 1 g of soil collected from sites close to various agriculturally used fields in the Münsterland region with 1 ml of CDC solution (1 mM; pH 8, set with NaOH) in a 2 ml plastic tube (Sarstedt, Nümbrecht, Germany). For each microcosm experiment, 30–60 plastic tubes were prepared in parallel and incubated at room temperature. At several time points, three tubes were used for sampling by centrifugation at >16,000 × *g* for 5 min at room temperature. Supernatants were stored at −20 °C until extraction for LC-MS measurements.

### Bile acid degradation in sand microcosms

Quartz sand (Siligran, washed; Ø: 0.7–1.2 mm) was obtained from Euroquarz (Dorsten, Germany) and sterilized by baking at 180 °C for at least 6 h. Sand microcosms were prepared by filling 3 g or 25 g of sand into petri dishes (Ø: 3.5 cm or 9.2 cm, respectively). To obtain cell suspensions for the sand microcosms, *E*. *coli* OP50_-ura_ and *P*. *stutzeri* Chol1 were cultivated overnight. Cell suspensions were prepared from re-fed cultures, containing *E*. *coli* OP50_-ura_ at an OD_600_ of 0.133 and *P*. *stutzeri* Chol1 at an OD_600_ of 0.013 or only *E*. *coli* OP50_-ura_ at an OD_600_ of 0.147. 0.25 mM, 0.5 mM or 1 mM of CDC was added to the cell suspensions as indicated. Petri dishes with a diameter of 9.2 cm or 3.5 cm were supplemented with 7.5 ml or 0.9 ml of cell suspension, respectively. Petri dishes with a diameter of 9.2 cm were used to study the degradation of CDC over 24 h. To maintain the sand/liquid ratio, samples containing cell suspension and sand were repeatedly withdrawn from the same petri dish, centrifuged at >16,000 × *g* for 5 min, with the supernatants used for HPLC-MS measurements. Petri dishes with a diameter of 3.5 cm were used to monitor the degradation of CDC over prolonged time periods and in the presence of 10 *C*. *elegans* non-synchronized adult worms on each petri dish. These petri dishes were incubated in darkness for up to five days at 21 °C and weighed every day to determine water loss. Freshly prepared cell suspensions were added daily to compensate for a loss of water and to provide fresh and active bacteria. At several time points, the supernatant of one petri dish each was withdrawn for LC-MS analysis.

### Fecundity of *C*. *elegans* in sand microcosms

Sand microcosms were prepared by filling 3 g of sterilized quartz sand into petri dishes (Ø: 3.5 cm). The water-holding capacity of the sand was determined at room temperature (~24%) and after two days at 60 °C (~35%). From these results, a mean value of 30% was chosen, and 900 μl of cell suspension (MMChol media with bacteria and, if applicable, with 0.25 mM of CDC) were added to the sand. Worms were fed with *E*. *coli* OP50_-ura_ and *P*. *stutzeri* Chol1. Preliminary experiments revealed a final *E*. *coli* OP50_-ura_ concentration of OD_600_ = 0.6 in the cell suspension as optimum feeding quantity because the reproduction of *C*. *elegans* over 4-day periods did not increase anymore with more *E*. *coli* OP50_-ura_ provided. As *P*. *stutzeri* Chol1 was applied at a final concentration of OD_600_ = 0.013, experiments without *P*. *stutzeri* Chol1 were carried out at a final *E*. *coli* OP50_-ura_ concentration of OD_600_ = 0.613. After inserting one *C*. *elegans* L4 worm in a liquid-filled gap in the sand sample, the samples were incubated for four days at 21 °C. 100 μl of fresh cell suspension with 1.25 mM of CDC, if applicable, were added daily.

After incubation, the offspring of the worm was extracted from sand using a modification of the centrifugal flotation method^84^ using Ludox (LUDOX® HS-40 colloidal silica; Sigma-Aldrich Chemie GmbH, Munich, Germany). 2 ml of a Ludox/M9 buffer solution (1:1) was pipetted onto the sand, which was slightly tilted. 4 × 500 μl of the liquid phase were then transferred to 4 Eppendorf tubes filled with 1 ml of M9 buffer^77^. These samples were centrifuged (2,700 × *g*, 1 min, 20 °C) and allowed to stand for 10 min. This extraction step was repeated another 3 times, resulting in 16 tubes. During the last application of the Ludox/M9 buffer, the sand was checked for worms. After transferring slightly more than 1 ml of the supernatants of the 16 samples into small petri dishes, living worms were counted. The remaining rests of the samples were united, resulting in 4 tubes. These samples were centrifuged and allowed to stand for at least 5 min. Using 1 ml of the supernatants, worms were again counted. After the addition of 1 ml of M9 buffer to each tube, centrifugation and a rest period of at least 5 min, the worms in 1 ml of the supernatants were again counted. The remaining rests of the 4 tubes were then transferred onto NGM plates, where worms were again counted. The effects of four different experimental conditions on the reproduction of *C*. *elegans* were tested in three independent experimental series. As the number of offspring varied considerably from series to series, the data of each experimental series were normalized (i.e., division by the mean number of offspring of each experimental series).

### Statistics

Data are given as means ± standard deviation (sd) or standard error (se). *n* indicates the number of biological replicates and *N* the number of animals used. SigmaPlot 11.0 (Systat Software, Erkrath, Germany) was used for graph preparation and statistical analysis. Statistical significances (*P* values) were calculated using one-, two-, or three-way analyses of variance (anova) and subsequent Student-Newman-Keuls analyses, chi-square analyses, or t-tests.

## Supplementary information

Supplemental Material

## Data Availability

The data analysed during this study are included in this published article (and its Supplementary Information files). The transcriptomic data are publically available from the NCBI Gene Expression Omnibus (GEO) under the accession number GSE126214.
