# Supplementary material for: Steroids originating from bacterial bile acid degradation affect Caenorhabditis elegans and indicate potential risks for the fauna of manured soils
Source: Sci Rep. 2019 Jul 31;9:11120. doi: 10.1038/s41598-019-47476-y (PMC6668416; doi:10.1038/s41598-019-47476-y)
Supplement: Supplementary file 1 — Supplemental Material [file 41598_2019_47476_MOESM1_ESM.pdf]

Steroids originating from bacterial bile acid degradation affect *Caenorhabditis elegans* and indicate potential risks for the fauna of manured soils

[+]M. N. Mendelski[1], [+]R. Dölling[1], [+]F. M. Feller[2], D. Hoffmann[1], L. Ramos Fangmeier[1], K. C. Ludwig[2,3], O. Yücel[2], A. Mährlein[2], R. J. Paul[1], B. Philipp[2,*]

[1]Institute of Zoophysiology, University of Münster (WWU), Münster, Germany

[2]Institute of Molecular Microbiology and Biotechnology, University of Münster (WWU), Münster, Germany

[3]Present address: Institute for Pharmaceutical Microbiology, University Hospital Bonn, University of Bonn, Bonn, Germany

[*]Corresponding author

Institute of Molecular Microbiology and Biotechnology, University of Münster (WWU), Münster, Germany

Correnssstrasse 3

48149 Münster

Phone: +251-83-39827

FAX: +251-83-38388

E-Mail: bodo.philipp@uni-muenster.de

[+]These authors contributed equally to this study.

Table S1. Gene ontology analyses. Gene ontology (GO) analyses (functional annotation chart; David Bioinformatics Resources 6.8) were carried out to determine the function of the groups of genes highlighted in Fig. 4 (red or green circles). The columns show (from left to right): **A**, annotation source (B, GOTERM_BP_Direct; I, Interpro; U, UP_Keywords); **T**, term (background colors indicate specific terms, *see* Results); **%**, the percentage share of the genes assigned to this term; **Q**, Q-value (Benjamini). Only dominant terms with minimal Q-values are shown.

Contrasts between 7α-HADD exposure and control conditions in wild type

FPKM_WT_7α-HADD vs. FPKM_WT_control (Red circles in Fig. 4a)
Genes: 160 identified, 3 non-identified

A	T	%	Q
U	Ribosomal protein	49.4	1.7E-128
U	Ribonucleoprotein	49.4	2.1E-120
B	Translation	49.4	7.4E-93
B	Reproduction	63.7	1.6E-41
B	Nematode larval development	61.3	1.7E-41
B	Embryo development ending in birth or egg hatching	68.1	7.2E-35
B	Apoptotic process	32.5	7.7E-34

Log2 (FPKM_WT_7α-HADD) vs. Log2 (FPKM_WT_control) (Green circles in Fig. 4b)
Genes: 1240 identified, 3 non-identified

A	T	%	Q
I	Domain of unknown function WSN	2.1	1.7E-15
B	Sensory perception of chemical stimulus	2.1	2.2E-03
B	Detection of chemical stimulus involved in sensory perception	2.9	1.9E-03
B	Protein phosphorylation	3.5	1.7E-03
B	Peptidyl-tyrosine dephosphorylation	1.3	3.4E-02
I	Protein-tyrosine phosphatase, receptor/non-receptor type	1.3	9.2E-02
I	Protein kinase-like domain	3.8	1.2E-01
I	Serine-threonine/tyrosine-protein kinase catalytic domain	1.4	2.5E-01
I	7TM GPCR, serpentine receptor class h (Srh)	2.2	2.2E-01
I	Protein kinase, catalytic domain	3.1	3.2E-01
B	G-protein coupled receptor signaling pathway	3.8	6.5E-01
B	Ion transport	2.3	7.2E-01

Log2 (FPKM_WT_7α-HADD) vs. Log2 (FPKM_WT_control) (Red circles in Fig. 4b)
Genes: 695 identified, 1 non-identified

A	T	%	Q
B	Detection of chemical stimulus involved in sensory perception	5.5	2.3E-16
I	7TM GPCR, serpentine receptor class h (Srh)	4.0	5.8E-07
B	Olfactory behavior	3.5	3.1E-07
B	G-protein coupled receptor signaling pathway	5.3	1.3E-06
B	Detection of chemical stimulus involved in sensory perception of smell	3.2	1.2E-06
I	7TM GPCR, serpentine receptor class r (Str)	3.0	1.8E-03
B	Sensory perception of chemical stimulus	1.7	3.6E-02
I	C-type lectin fold	2.6	2.1E-01
I	C-type lectin	2.0	6.4E-01
I	C-type lectin-like	2.2	5.7E-01

Contrasts between *nhr-69*Δ and wild type under control conditions

FPKM_*nhr-69*Δ_control vs. FPKM_WT_control (Red circles in Fig. 4e)
Genes: 160 identified, 3 non-identified

A	T	%	Q
U	Ribosomal protein	49.4	1.7E-128
U	Ribonucleoprotein	49.4	2.1E-120
B	Translation	49.4	7.4E-93
B	Reproduction	63.7	1.6E-41

Log2 (FPKM_*nhr-69*Δ_control) vs. Log2 (FPKM_WT_control) (Green circles in Fig. 4f)
Genes: 3147 identified, 6 non-identified

A	T	%	Q
I	P-loop containing nucleoside triphosphate hydrolase	4.6	2.0E-09
B	Protein phosphorylation	3.5	6.9E-06
B	Intracellular signal transduction	1.6	8.8E-06
I	Armadillo-type fold	1.9	1.2E-04

Log2 (FPKM_*nhr-69*Δ_control) vs. Log2 (FPKM_WT_control) (Red circles in Fig. 4f)
Genes: 499 identified, 3 non-identified

A	T	%	Q
B	Detection of chemical stimulus involved in sensory perception	5.2	8.1E-10
I	Histone-fold	3.0	7.2E-07
B	G-protein coupled receptor signaling pathway	6.2	1.6E-06
B	Detection of chemical stimulus involved in	3.8	1.3E-06

A	T	%	Q	A	T	%	Q	A	T	%	Q
									sensory perception of smell		
B	Nematode larval development	61.3	1.7E-41	I	Protein kinase-like domain	3.7	2.0E-04	I	Histone core	2.4	3.1E-06
B	Embryo development ending in birth or egg hatching	68.1	7.2E-35	I	Protein kinase, catalytic domain	3.3	2.5E-04	B	Olfactory behavior	3.8	2.7E-06
B	Apoptotic process	32.5	7.7E-34	B	Ion transport	2.7	3.9E-03	I	7TM GPCR, serpentine receptor class h (Srh)	4.0	3.2E-05
				B	Sensory perception of chemical stimulus	1.6	1.0E-02	I	7TM GPCR, serpentine receptor class r (Str)	3.4	8.8E-04
				B	Signal transduction	2.9	2.0E-02	I	C-type lectin-like	3.4	2.1E-03
				I	Domain of unknown function DUF38, Caenorhabditis species	1.7	1.0E-01	I	C-type lectin	3.2	1.8E-03
				B	G-protein coupled receptor signaling pathway	4.1	1.8E-01	I	C-type lectin fold	3.4	3.9E-03
				B	Detection of chemical stimulus involved in sensory perception	2.1	6.2E-01	B	Sensory perception of chemical stimulus	2.0	3.8E-02
				B	Transport	4.6	5.7E-01				
				B	Metabolic process	5.8	7.2E-01				
				B	Phosphorylation	2.2	6.9E-01				
				I	Protein kinase, ATP binding site	1.8	9.3E-01				
				B	Olfactory behavior	1.7	9.8E-01				
				B	Sterol hormone mediated signaling pathway	1.7	9.8E-01				

Contrasts between *nhr-69*Δ and wild type under 7α-HADD exposure

FPKM_*nhr-69*Δ_7α-HADD vs. FPKM_WT_7α-HADD (Red circles in Fig. 4g) Genes: 145 identified, 4 non-identified				Log_2 (FPKM_*nhr-69*Δ_7α-HADD) vs. Log_2 (FPKM_WT_7α-HADD) (Green circles in Fig. 4h) Genes: 1684 identified, 4 non-identified				Log_2 (FPKM_*nhr-69*Δ_7α-HADD) vs. Log_2 (FPKM_WT_7α-HADD) (Red circles in Fig. 4h) Genes: 494 identified, 2 non-identified			
A	T	%	Q	A	T	%	Q	A	T	%	Q
U	Ribosomal protein	53.8	9.8E-131	B	Detection of chemical stimulus involved in sensory perception	3.8	1.8E-13	B	Detection of chemical stimulus involved in sensory perception	5.9	5.4E-12
U	Ribonucleoprotein	53.8	8.9E-123	B	G-protein coupled receptor signaling pathway	5.4	4.6E-10	I	7TM GPCR, serpentine receptor class h (Srh)	4.3	8.8E-06
B	Translation	53.8	3.0E-94	B	Olfactory behavior	2.9	3.7E-08	B	Sensory perception of chemical stimulus	2.6	1.4E-03
B	Reproduction	66.9	1.8E-40	B	Detection of chemical stimulus involved in sensory perception of smell	2.7	3.1E-08	B	G-protein coupled receptor signaling pathway	5.1	2.3E-03
B	Nematode larval development	62.1	5.5E-37	I	Domain of unknown function DUF38, Caenorhabditis species	2.8	2.1E-07	I	GPCR, rhodopsin-like, 7TM	4.0	6.1E-02
B	Embryo development ending in birth or egg hatching	73.1	9.8E-37	B	Sensory perception of chemical stimulus	2.1	4.8E-07	B	Detection of chemical stimulus involved in sensory perception of smell	2.0	3.5E-01
B	Apoptotic process	33.8	5.5E-32	I	7TM GPCR, serpentine receptor class r (Str)	2.5	1.2E-04	B	Olfactory behavior	2.0	4.0E-01
				I	7TM GPCR, serpentine receptor class h (Srh)	2.4	8.2E-04	I	C-type lectin-like	2.2	7.7E-01
				B	Sterol hormone mediated signaling pathway	2.1	1.1E-02	I	C-type lectin	2.0	7.6E-01
								I	7TM GPCR, serpentine receptor class r (Str)	2.0	7.1E-01

I	C-type lectin fold	2.2	7.0E-01
B	Regulation of transcription, DNA-templated	3.6	6.7E-01

Table S2. Gene ontology analyses. Gene ontology (GO) analyses (functional annotation chart; David Bioinformatics Resources 6.8) were carried out to determine the function of the groups of DEGs highlighted in Fig. S2 (red or green circles). The columns show (from left to right): **A**, annotation source (B, GOTERM_BP_Direct; U, UP_Keywords); **T**, term (background colors indicate specific terms, *see* Results); **%**, the percentage share of the DEGs assigned to this term; **Q**, Q-value (Benjamini). Only dominant terms with minimal Q-values are shown.

Contrasts between 7α-HADD exposure and control conditions in wild type

FPKM_WT_7α-HADD vs. FPKM_WT_control (Red circles in Fig. S2a) Genes: 101 identified				Log_2 (FPKM_WT_7α-HADD) vs. Log_2 (FPKM_WT_control) (Green circles in Fig. S2b) Genes: 256 identified				Log_2 (FPKM_WT_7α-HADD) vs. Log_2 (FPKM_WT_control) (Red circles in Fig. S2b) Genes: 60 identified, 1 non-identified			
A	T	%	Q	A	T	%	Q	A	T	%	Q
U	Ribosomal protein	54	1.7E-89	U	Coiled coil	41	7.7E-48	U	Signal	48	2.5E-05
U	Ribonucleoprotein	54	1.4E-84	B	Embryo development ending in birth or egg hatching	47	2.7E-17				
B	Translation	52	1.1E-62	B	Reproduction	36	5.1E-14				
B	Nematode larval development	61	4.6E-26	U	ATP-binding	15	4.4E-13				
B	Reproduction	62	8.5E-25	U	Nucleotide-binding	16	3.9E-11				
B	Apoptotic process	30	8.9E-18	B	Nematode larval development	31	2.7E-10				
B	Embryo development ending in birth or egg hatching	62	6.9E-17	U	Nucleus	17	2.7E-09				
U	Collagen	13	1.3E-09	B	Hermaphrodite genitalia development	16	1.3E-08				
B	Cytoplasmic translation	7.9	2.2E-09	B	Body morphogenesis	14	4.4E-08				
U	RNA-binding	12	7.3E-09	B	DNA repair	6.3	5.7E-07				
B	Molting cycle, collagen and cuticulin-based cuticle	17	9.9E-09	B	Gonad development	9.8	1.3E-06				
B	Ribosomal large subunit assembly	5.9	3.3E-06	U	Helicase	4.3	1.1E-06				
U	rRNA-binding	5	3.6E-06	B	Receptor-mediated endocytosis	14	2.7E-06				

Contrasts between *nhr-69*Δ and wild type under control conditions

FPKM_*nhr-69*Δ_control vs. FPKM_WT_control (Red circles in Fig. S2c) Genes: 151 identified, 1 non-identified				Log_2 (FPKM_*nhr-69*Δ_control) vs. Log_2 (FPKM_WT_control) (Green circles in Fig. S2d) Genes: 2478 identified, 1 non-identified				Log_2 (FPKM_*nhr-69*Δ_control) vs. Log_2 (FPKM_WT_control) (Red circles in Fig. S2d) Genes: 501 identified, 2 non-identified			
A	T	%	Q	A	T	%	Q	A	T	%	Q
U	Ribosomal protein	52	3.0E-131	U	Coiled coil	22	1.9E-135	U	Nucleosome core	5.4	2.6E-21
U	Ribonucleoprotein	52	3.8E-123	U	ATP-binding	8.8	2.3E-42	U	Methylation	4.6	2.8E-16
B	Translation	52	4.9E-95	U	Alternative splicing	9	2.1E-39	B	Embryo development ending in birth or egg hatching	29	4.1E-14
B	Nematode larval development	62	1.1E-39	U	Nucleotide-binding	9.2	6.7E-33	U	Chromosome	5.4	1.6E-14
B	Reproduction	64	3.8E-38	U	Metal-binding	11	3.3E-23	U	Acetylation	4.4	7.0E-12
B	Embryo development ending in birth or egg hatching	68	4.8E-32	U	Nucleus	10	3.1E-19	B	Nucleosome assembly	3.8	1.1E-10
B	Apoptotic process	32	2.0E-31	U	Zinc	7.3	1.8E-17	B	Reproduction	21	1.8E-09

A	T	%	Q	A	T	%	Q	A	T	%	Q
B	Cytoplasmic translation	9.3	1.9E-19	U	Developmental protein	4.6	5.9E-17	U	Isopeptide bond	3	7.4E-10
U	rRNA-binding	5.3	2.2E-11	U	Hydrolase	7.2	2.9E-16	U	Ribonucleoprotein	5	6.4E-10
B	Ribosomal small subunit assembly	6	3.9E-11	B	Reproduction	19	1.2E-12	B	Nematode larval development	18	5.6E-07
B	Molting cycle, collagen and cuticulin-based cuticle	15	7.2E-11	U	Zinc-finger	5.5	8.4E-14	U	Mitochondrion	5	1.7E-07
U	RNA-binding	9.3	4.6E-09	B	Locomotion	13	9.2E-12	U	Ubl conjugation	2.6	8.2E-07
B	Ribosomal large subunit assembly	5.3	2.1E-08	U	Helicase	1.5	2.1E-12				
				U	Kinase	3.9	2.2E-12				

Contrasts between *nhr-69*Δ and wild type under 7α-HADD exposure

FPKM_*nhr-69*Δ_7α-HADD vs. FPKM_WT_7α-HADD (Red circles in Fig. S2e) Genes: 134 identified				Log_2 (FPKM_*nhr-69*Δ_7α-HADD) vs. Log_2 (FPKM_WT_7α-HADD) (Green circles in Fig. S2f) Genes: 1437 identified, 2 non-identified				Log_2 (FPKM_*nhr-69*Δ_7α-HADD) vs. Log_2 (FPKM_WT_7α-HADD) (Red circles in Fig. S2f) Genes: 173 identified, 1 non-identified			
A	T	%	Q	A	T	%	Q	A	T	%	Q
U	Ribosomal protein	58	1.3E-134	U	Coiled coil	24	3.1E-86	U	Acetylation	14	8.1E-26
U	Ribonucleoprotein	58	1.2E-126	U	ATP-binding	9	1.3E-23	U	Nucleosome core	10	1.3E-18
B	Translation	58	6.3E-98	U	Nucleotide-binding	9.3	5.8E-18	U	Collagen	13	3.8E-17
B	Nematode larval development	66	8.3E-40	U	Metal-binding	11	2.7E-12	U	Chromosome	11	1.8E-15
B	Reproduction	69	1.2E-39	U	Zinc	7.7	3.2E-11	U	Isopeptide bond	6.9	8.4E-12
B	Embryo development ending in birth or egg hatching	74	1.8E-34	U	Helicase	1.9	3.0E-10	U	Methylation	7.5	1.6E-11
B	Apoptotic process	36	1.6E-32	U	Zinc-finger	5.8	3.8E-09	U	Ubl conjugation	6.9	5.4E-11
B	Cytoplasmic translation	10	4.5E-20	U	Alternative splicing	6.9	1.6E-08	B	Nucleosome assembly	6.9	5.8E-10
U	rRNA-binding	6	8.4E-12	U	Nucleus	9.4	7.4E-08	U	Cell projection	7.5	1.2E-08
B	Molting cycle, collagen and cuticulin-based cuticle	17	9.6E-12	U	DNA damage	1.4	7.8E-08	B	Chromatin silencing	4	2.7E-07
U	RNA-binding	11	5.6E-11	U	DNA repair	1.3	4.4E-07	B	Pharynx development	4.6	2.1E-06
B	Ribosomal small subunit assembly	6	1.4E-09	B	DNA repair	2.2	4.4E-05	B	Innate immune response	8.1	8.9E-06
B	Ribosomal large subunit assembly	6	9.2E-09								

Table S3. Significant differences (*P*-values) in the number of offspring (N) determined over all three generations (F0, F1, F2) between test (i.e. steroid application) and control conditions (N_{F0F1F2}_test vs. N_{F0F1F2}_control) or intergenerational differences in the number of offspring (N_{F0} vs. N_{F1}, N_{F0} vs. N_{F2}, or N_{F1} vs. N_{F2}) were ascertained in wild type and *nhr-69*Δ by three-way ANOVA and subsequent Student-Newman-Keuls analysis. Significant differences are indicated by white background colours.

Strain	N_{F0F1F2}_test vs. N_{F0F1F2}_control		N_{F0} vs. N_{F1}	N_{F0} vs. N_{F2}	N_{F1} vs. N_{F2}
	Tested steroid	*P*-value	*P*-value	*P*-value	*P*-value
wild type	testosterone	< 0.001	0.105 (no intergenerational differences)		
	ADD	0.009	0.309 (no intergenerational differences)		
	7α-HADD	< 0.001	0.165	< 0.001	< 0.001
	12β-HADD	< 0.001	0.380	0.007	0.008
	12β-DHADD	0.014	0.255 (no intergenerational differences)		
*nhr-69*Δ	testosterone	0.937	0.323 (no intergenerational differences)		
	ADD	0.707	0.304 (no intergenerational differences)		
	7α-HADD	0.086	0.082	0.015	0.093
	12β-HADD	0.718	0.073	0.065	0.011
	12β-DHADD	0.927	0.094 (no intergenerational differences)		

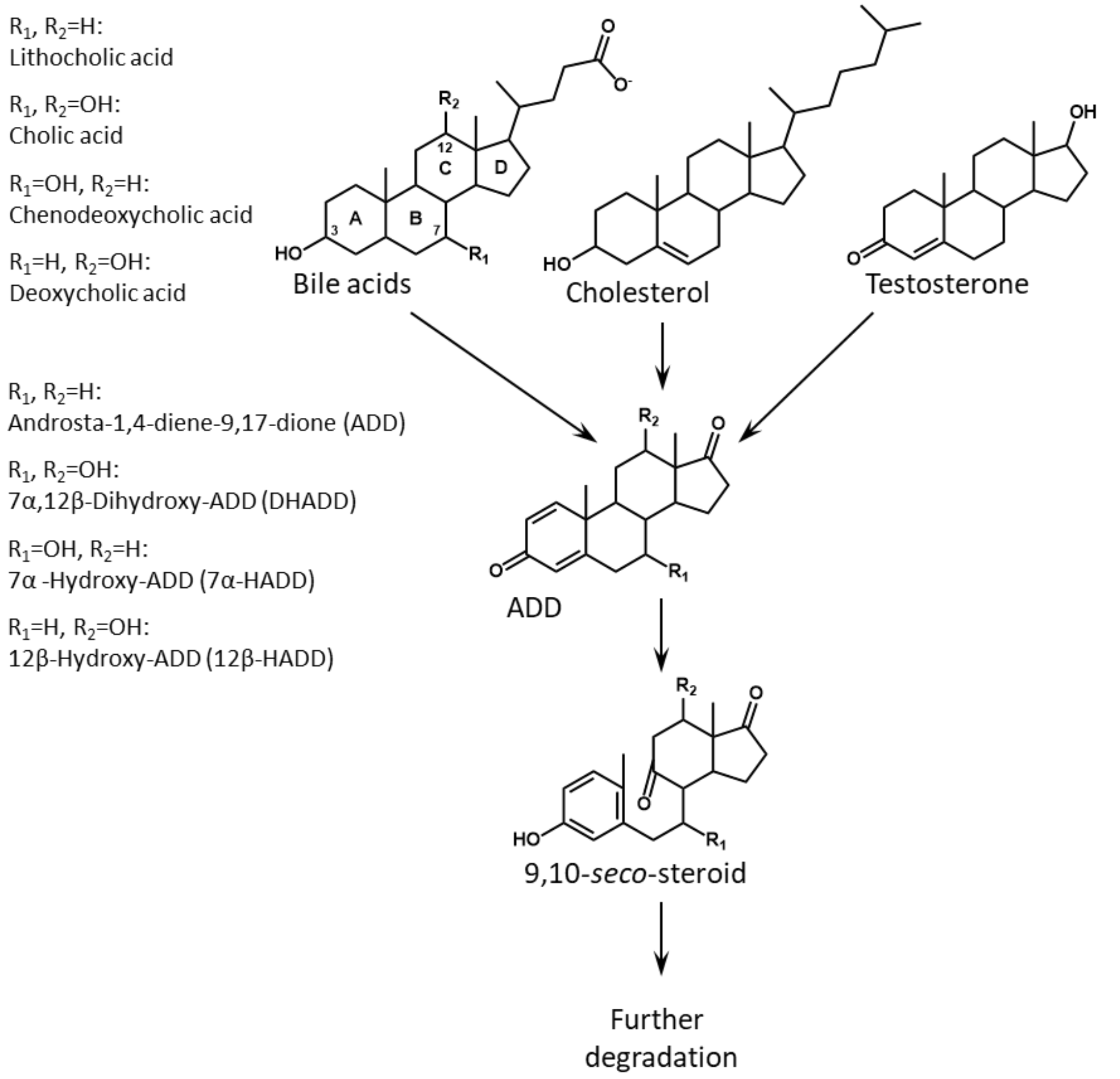

Fig. S1. Scheme of steroid degradation via ADDs and 9,10-*seco*-pathway. Many steroids such as bile acids, sterols and steroid hormones are degraded via ADDs.

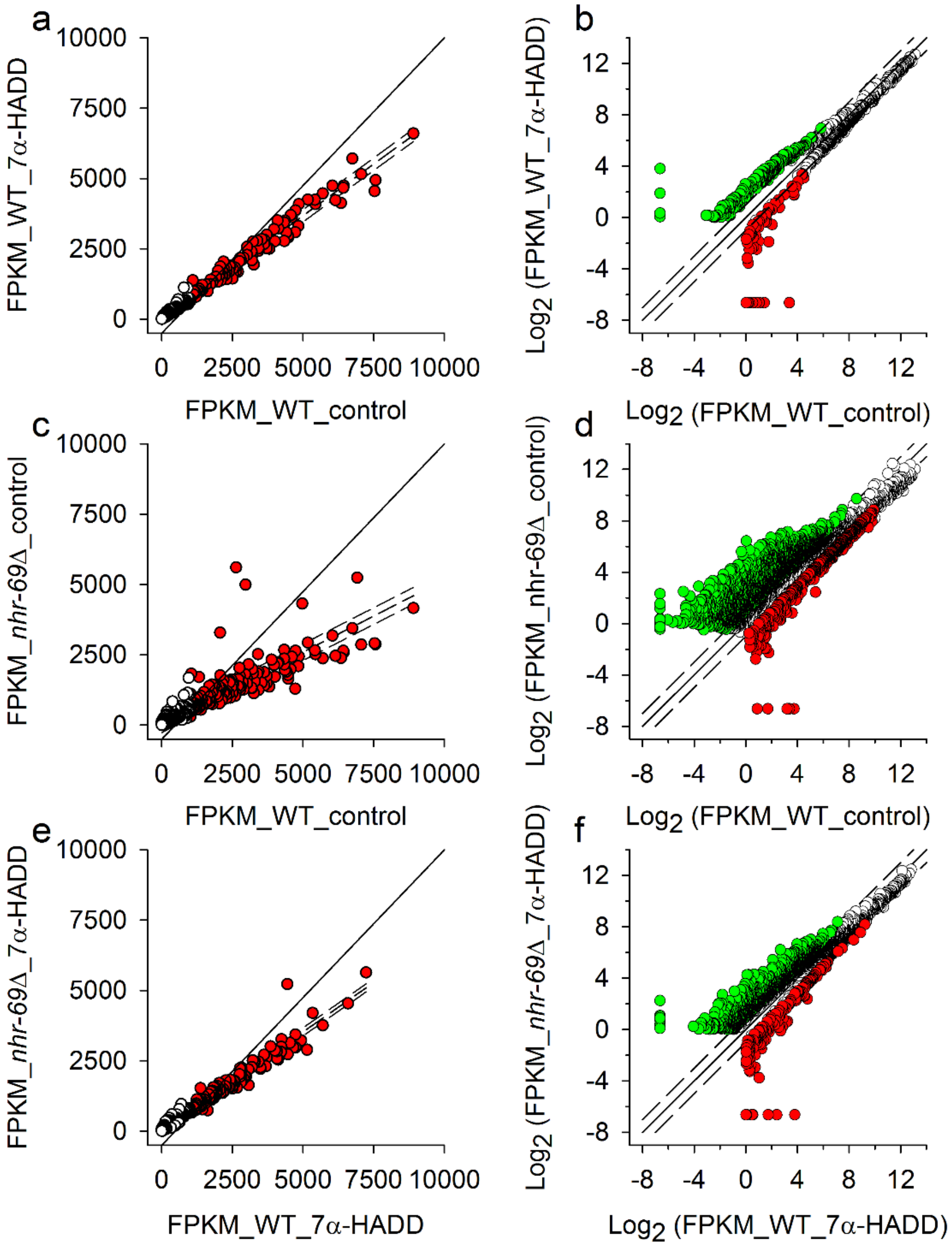

Fig. S2. Expression intensities of DEGs in wild type or *nhr-69*Δ after breeding under control or test conditions.

To analyze in the transcriptomic data (*see* Fig. 4) the regulation of differentially expressed genes with a diverge probability of greater than or equal to 0.8 (DEGs), the mean expression intensities (averaged FPKM values) of corresponding DEGs (from different conditions or strains under equal conditions) were plotted against each other [double linear (left graphs) or double $\log_2$ (right graphs) x-y plots] in case of (**a**, **b**) wild type under control and test (7α-HADD exposure) conditions or wild type and *nhr-69*Δ under (**c**, **d**) control or (**e**, **f**) test conditions (continuous lines mark the 45-degree diagonal lines). DEGs with a mean FPKM value higher than 1000 on the x-axis deviated from the 45-degree diagonal line (left graphs, red circles and dashed linear regression and 99% prediction lines). DEGs with lower mean FPKM values and deviations from equal $\log_2$-fold expression intensity (45-degree diagonal lines) of lesser than -1 or greater than 1 between x-axis and y-axis (right graphs, green or red circles and dashed offsets of -1 and 1) were also functionally characterized by GO analyses (see Supplemental Table S2).

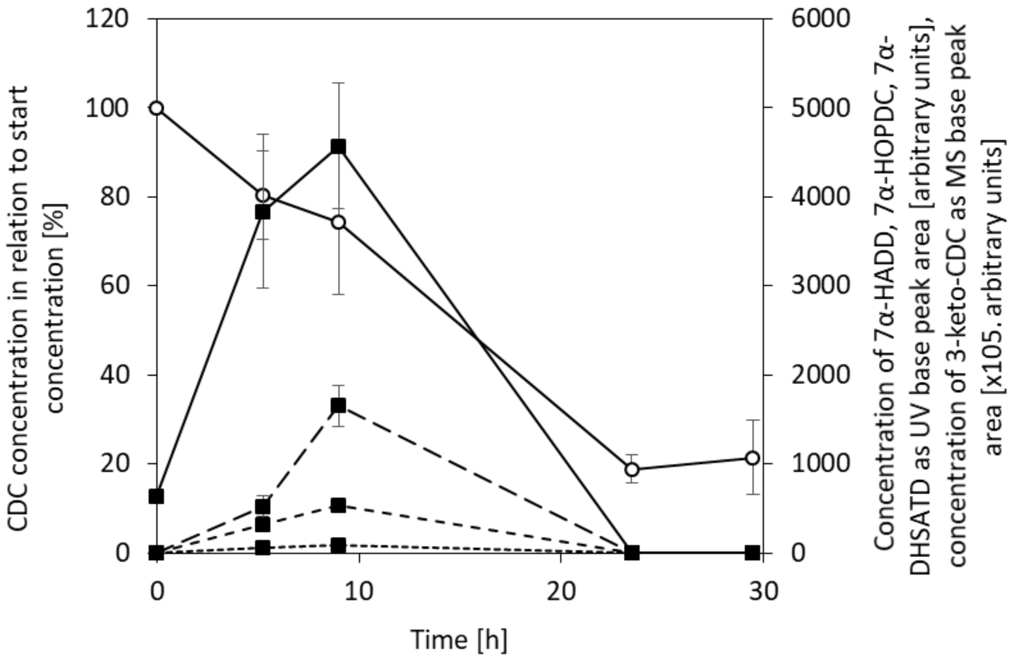

Fig. S3. Degradation of 0.25 mM CDC (open circles) and accumulation of intermediates (closed squares, 3-keto-CDC: continuous line, 7α-HOPDC: little dots, 7α-HADD: broken line, 7α-DHSATD: big dots) in sand environment by *P. stutzeri* Chol1 and in presence of *E. coli* OP50$_{\text{-ura}}$. *P. stutzeri* Chol1 and *E. coli* OP50$_{\text{-ura}}$ were grown and reactivated with CDC or LB, respectively, and reactivated in the late exponential growth phase. 900 µl cell suspension containing *P. stutzeri* Chol1 at an OD_{600} of 0.013 and *E. coli* OP50$_{\text{-ura}}$ at an OD_{600} of 0.147 and supplemented with 0.25 mM CDC were dispensed on 3 g quartz sand in petri dishes with 3.5 cm diameter. Petri dishes were incubated at 21°C. For sampling, cell suspension and sand were taken and centrifuged. The supernatant was analysed by LC-MS. CDC concentration was determined as base peak area of total ion counts in negative mode MS measurements and is shown in relation to the start concentration. Concentration of 3-keto-CDC was determined as base peak area of total ion counts in negative mode MS measurements. Concentration of 7α-HADD and 7α-HOPDC were determined as base peak areas in UV chromatograms at an analysis wavelength of 245 nm and 7α-DHSATD at a wavelength of 280 nm. (mean ± sd, *n* = 3).

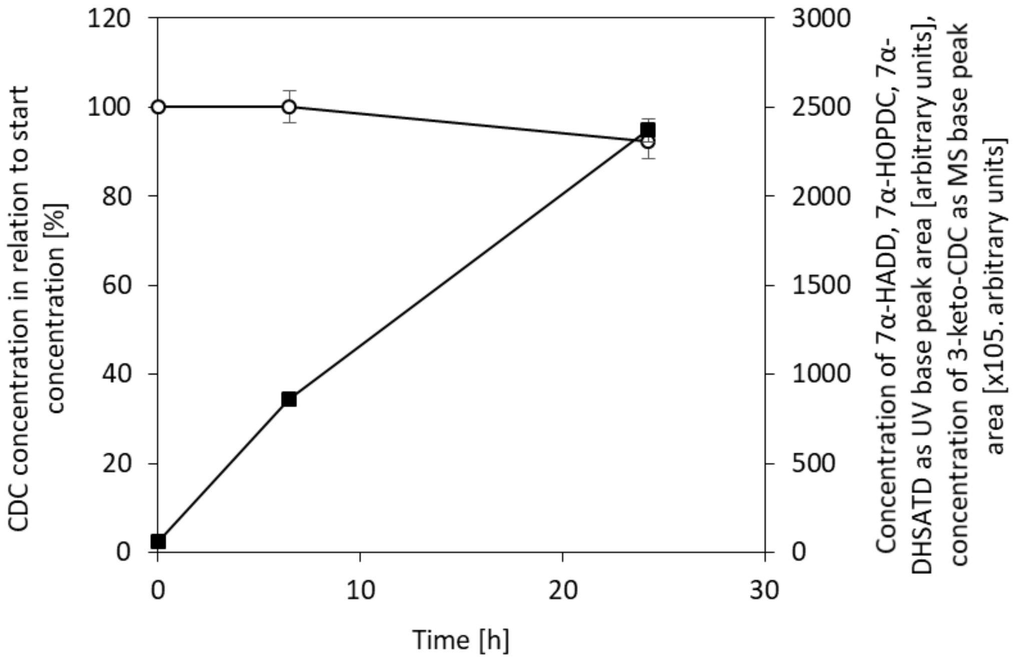

Fig. S4. Degradation of 0.25 mM CDC (open circles) and accumulation of 7-keto-CDC (closed squares) in sand environment by *E. coli* OP50$_{\text{-ura}}$. *E. coli* OP50$_{\text{-ura}}$ was grown and reactivated with LB and reactivated in the late exponential growth phase. 900 µl cell suspension containing *E. coli* OP50$_{\text{-ura}}$ at an OD_{600} of 0.147 and supplemented with 0.25 mM CDC were dispensed on 3 g quartz sand in petri dishes with 3.5 cm diameter. Petri dishes were incubated at 21°C. For sampling, cell suspension and sand were taken and centrifuged. The supernatant was analysed by LC-MS. CDC concentration was determined as base peak area of total ion counts in negative mode MS measurements and is shown in relation to the start concentration. Concentration of 3-keto-CDC was determined as base peak area of total ion counts in negative mode MS measurements. (mean ± sd, *n* = 3).

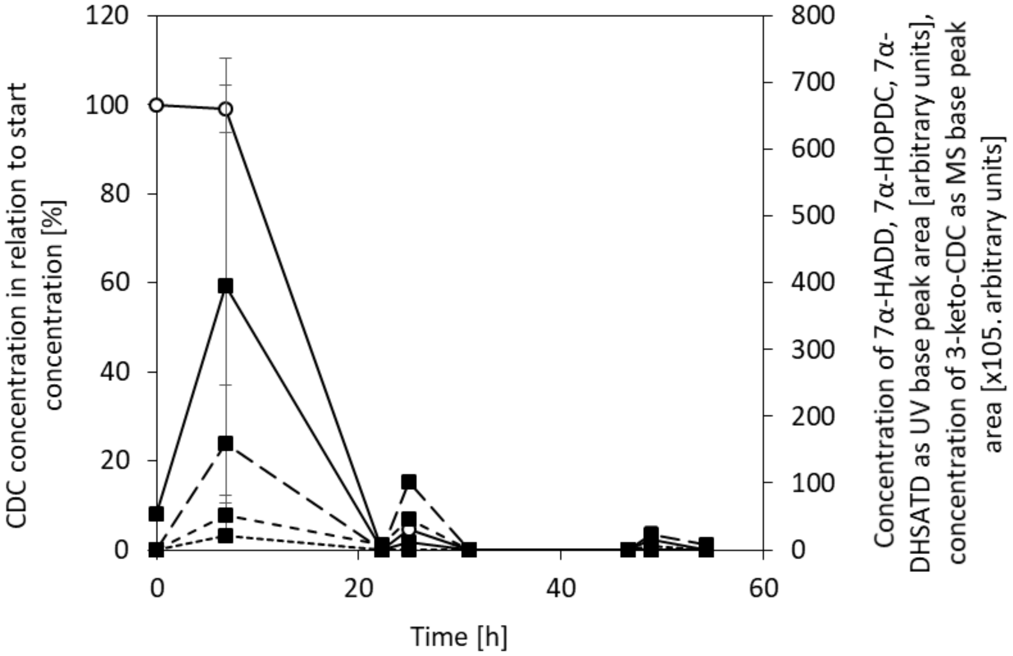

Fig. S5. Degradation of 0.25 mM CDC (open circles) and accumulation of intermediates (closed squares, 3-keto-CDC: continuous line, 7α-HOPDC: little dots, 7α-HADD: broken line, 7α-DHSATD: big dots) in sand environment by *P. stutzeri* Chol1 in presence of *E. coli* OP50$_{\text{-ura}}$ and *C. elegans*. *P. stutzeri* Chol1 and *E. coli* OP50$_{\text{-ura}}$ were grown and reactivated with CDC or LB, respectively, and reactivated in the late exponential growth phase. 900 µl cell suspension containing *P. stutzeri* Chol1 at an OD_{600} of 0.013 and *E. coli* OP50$_{\text{-ura}}$ at an OD_{600} of 0.147 and supplemented with 0.25 mM CDC were dispensed on 3 g quartz sand in petri dishes with 3.5 cm diameter. Ten non-synchronized adult worms of *C. elegans* were placed on each Petri dish. Petri dishes were incubated at 21°C. For sampling, cell suspension and sand were taken and centrifuged. The supernatant was acidified, extracted with ethyl acetate, resolved in methanol and finally analysed by LC-MS. CDC concentration was determined as base peak area of total ion counts in negative mode MS measurements and is shown in relation to the start concentration. Concentration of 3-keto-CDC was determined as base peak area of total ion counts in negative mode MS measurements. Concentration of 7α-HADD and 7α-HOPDC were determined as base peak areas in UV chromatograms at an analysis wavelength of 245 nm and 7α-DHSATD at a wavelength of 280 nm (mean ± sd, *n* = 3).
